# Difficulty in inferring microbial community structure based on co-occurrence network approaches

**DOI:** 10.1186/s12859-019-2915-1

**Published:** 2019-06-13

**Authors:** Hokuto Hirano, Kazuhiro Takemoto

**Affiliations:** Department of Bioscience and Bioinformatics, Kyushu Institute of Technology, Iizuka, Fukuoka 820-8502 Japan

**Keywords:** Microbiome, Correlation network analysis, Microbial ecology, Complex networks

## Abstract

**Background:**

Co-occurrence networks—ecological associations between sampled populations of microbial communities inferred from taxonomic composition data obtained from high-throughput sequencing techniques—are widely used in microbial ecology. Several co-occurrence network methods have been proposed. Co-occurrence network methods only infer ecological associations and are often used to discuss species interactions. However, validity of this application of co-occurrence network methods is currently debated. In particular, they simply evaluate using parametric statistical models, even though microbial compositions are determined through population dynamics.

**Results:**

We comprehensively evaluated the validity of common methods for inferring microbial ecological networks through realistic simulations. We evaluated how correctly nine widely used methods describe interaction patterns in ecological communities. Contrary to previous studies, the performance of the co-occurrence network methods on compositional data was almost equal to or less than that of classical methods (e.g., Pearson’s correlation). The methods described the interaction patterns in dense and/or heterogeneous networks rather inadequately. Co-occurrence network performance also depended upon interaction types; specifically, the interaction patterns in competitive communities were relatively accurately predicted while those in predator–prey (parasitic) communities were relatively inadequately predicted.

**Conclusions:**

Our findings indicated that co-occurrence network approaches may be insufficient in interpreting species interactions in microbiome studies. However, the results do not diminish the importance of these approaches. Rather, they highlight the need for further careful evaluation of the validity of these much-used methods and the development of more suitable methods for inferring microbial ecological networks.

**Electronic supplementary material:**

The online version of this article (10.1186/s12859-019-2915-1) contains supplementary material, which is available to authorized users.

## Background

Many microbes engage with one another through interspecific interactions (e.g., mutualistic and competitive interactions) to compose ecological communities and interrelate with their surrounding environments (e.g., their hosts) [1]. Investigating such communities is important not only in the context of basic scientific research [2, 3], but also in applied biological research fields, such as in medical [4] and environmental sciences [5]. Remarkable development of high-throughput sequencing techniques—e.g., 16S ribosomal RNA gene sequencing and metagenomics as well as computational pipelines—have provided snapshots of taxonomic compositions in microbial communities across diverse ecosystems [6] and revealed that microbial compositions are associated with human health and ecological environments. For example, microbial composition in the human gut is interrelated with by numerous diseases—such as diabetes and cardiovascular disease—age, diet, and antibiotic use [7, 8]. The composition of soil microbial communities is related to climate, aridity, pH, and plant productivity [9]. However, previous studies have been limited to the context of species composition, and the effect of the structure of microbial communities (microbial ecological networks) on such associations is unclear due to a lack of reliable methods through which real interaction networks can be captured. Thus, co-occurrence networks, which infer ecological associations between sampled populations of microbial communities obtained from high-throughput sequencing techniques, have been attracting attention [10]. Co-occurrence network approaches are also related to weighted correlation network analyses [11–13] for inferring molecular networks from high-throughput experimental data, such as gene expression data. A number of methods for inferring microbial association have been proposed.

As a simple metric, Pearson’s correlation coefficient is considered. Additionally, Spearman’s correlation coefficient and maximal information coefficient (MIC) [14] are useful for accurately detecting non-linear associations. However, these metrics may not be applicable to compositional data because the assumption of independent variables may not be satisfied due to the constant sum constraint [15]. Particularly, spurious correlations may be observed when directly applying these metrics to compositional data. To avoid this limitation, Sparse Correlations for Compositional data (SparCC) [16] has been developed. SparCC is an iterative approximation approach and estimates the correlations between the underlying absolute abundances using the log-ratio transformation of compositional data under the assumptions that real-world microbial networks are large-scale and sparse. However, SparCC is not efficient due to its high computational complexity. Thus, regularized estimation of the basis covariance based on compositional data (REBACCA) [17] and correlation inference for compositional data through Lasso (CCLasso) [18] have been proposed. These methods are considerably faster than SparCC by using the *l*_1_-norm shrinkage method (i.e., least absolute shrinkage and selection operator; Lasso). SparCC has further limitations, as it does not consider errors in compositional data and the inferred covariance matrix may be not positive definite. To avoid these limitations, CCLasso considers a loss function inspired by the lasso penalized *D*-trace loss.

However, correlation-based approaches such as those mentioned above may detect indirect associations. To differentiate direct and indirect interactions in correlation inference, other methods have been developed. In this context, inverse covariance matrix-based approaches are often used because they estimate an underlying graphical model, employing the concept of conditional independence. Typically, Pearson’s and Spearman’s partial correlation coefficients are used [19]; however, they may be not applicable to compositional data because statistical artifacts may occur due to the constant sum constraint. Thus, SParse InversE Covariance Estimation for Ecological ASsociation Inference (SPIEC-EASI) was proposed [20]. It infers an ecological network (inverse covariance matrix) from compositional data using the log-ratio transformation and sparse neighborhood selection.

These inference methods have been implemented as software packages and applied in several microbial ecology studies, such as investigations of human [21–24] and soil microbiomes [25–27]. While these methods only infer ecological associations, they are often used for discussing biological insights into interspecies interactions (i.e., microbial ecological networks [28]).

Nevertheless, further careful examination may be required to determine the importance of co-occurrence network approaches. The validity of these inference methods is still debatable [29] because they simply employ parametric statistical models, although microbial abundances are determined through population dynamics [2, 3]. Berry and Widder [30] used a mathematical model to determine population dynamics, generating (relative) abundance data based on population dynamics on an interaction pattern (network structure), and evaluated how correctly correlation-based methods reproduce the original interaction pattern. In particular, detecting interactions was harder for larger and/or more heterogeneous networks. However, they only compared earlier methods (e.g., Pearson’s correlation and SparCC) and not later methods (e.g., CCLasso) and the graphical model-based methods. In addition, whether further examination and comparison of performance is required remains debatable, since arbitrary thresholds were used to calculate sensitivity and specificity. Moreover, the effects of interaction type, such as mutualism or competition, on co-occurrence network performance were poorly considered, even though pairs of species exhibit well-defined interactions in natural systems [31]. Weiss et al. [10] considered interaction types and evaluated correlation-based methods using a population dynamics model; however, they only examined small-scale (up to six species) networks due to system complexity, although compositional-data methods (e.g., SparCC) assume large-scale networks. Furthermore, graphical model-based methods were not evaluated.

We comprehensively evaluated the validity of both correlation-based and graphical model-based methods for inferring microbial ecological networks. In particular, we focused on nine widely used methods. Following previous studies [10, 30], we generated relative abundance (compositional) data using a dynamical model with network structure and evaluated how accurately these methods recapitulate the network structure. We show that the performance of later methods was almost equal to or less than that of classical methods, contrary to previous studies. Moreover, we also demonstrate that co-occurrence network performance depends upon interaction types.

## Methods

### Generation of relative abundance data using a dynamical model

Following [30], we used the *n*-species generalized Lotka–Volterra (GLV) equation to generate abundance data:

$$ \frac{\mathrm{d}}{\mathrm{d}t}{N}_i(t)={N}_i(t)\left({r}_i+\sum \limits_{j=1}^n{M}_{ij}{N}_j(t)\right), $$

where *N*_*i*_(*t*) and *r*_*i*_ correspond to the abundance of species *i* at time *t* and the growth rate of species *i*, respectively. *M*_*ij*_ is an interaction matrix and indicates the contribution of species *j* to the growth of species *i*. In particular, *M*_*ij*_ was determined by considering network structure and interaction types; the diagonal elements *M*_*ii*_ in the interaction matrices, representing self-regulation, were set to − 1. Unlike a similar model used in a previous study [30], the carrying capacity of each species is set to be equivalent to its growth rate for simplicity.

To generate *M*_*ij*_, we first produce undirected networks with *n* nodes and average degree 〈*k*〉 = 2*m*/*n*, where *n* indicate the number of species and *m* is the number of edges. This is done by generating adjacency matrices *A*_*ij*_ using models for generating networks. Following Layeghifard et al. [28], three types of network structure were considered: random networks, small-world networks, and scale-free networks. In all cases *A*_*ij*_ = 1 if node (species) *i* interacts with node (species) *j* and *A*_*ij*_ = 0, otherwise, and *A*_*ij*_ = *A*_*ji*_ to have undirected networks.

The Erdős–Rényi model [32] was used to generate random networks in which the node degree follows a Poisson distribution where the mean is 〈*k*〉. The model networks are generated by drawing edges between *m* (=*n*〈*k*〉/2) node pairs that were randomly selected from the set of all possible node pairs. Specifically, we used *erdos.renyi.game* in the *igraph* package (version 1.2.2) of R (version 3.5.1; www.r-project.org), with the argument *type = “gnm”*.

However, real-world networks, including microbial ecological networks, are not random; instead, they are clustered (compartmentalized) and heterogeneous [28, 32–34].

The Watts–Strogatz model [35] was used to generate small-world networks whose clustering coefficients are higher than expected and random. The model networks are generated by randomly rewiring ⌊*p*_WS_*m* + 0.5⌋ edges in a one-dimensional lattice where *p*_WS_ corresponds to the rewiring probability (ratio) ranging within [0,1]. Specifically, we used the *sample_smallworld* function in the *igraph* package; *p*_WS_ was set to 0.05.

The Chung–Lu model [36] was used to generate scale-free networks in which the degree distributions are heterogeneous. In the model, *m* (=*n*〈*k*〉/2) edges are drawn between randomly selected nodes according to node weight (*i* + *i*_0_ − 1)^*ξ*^ where *ξ* ∈ [0, 1] and *i* denotes the node index (i.e., *i* = 1, …, *n*) and the constant *i*_0_ is considered to eliminate the finite-size effects [37]. A generated network shows that *P*(*k*) ∝ *k*^−*γ*^, where *γ* = 1 + 1/*ξ* [36, 37] and *P*(*k*) is the degree distribution. Specifically, we used the *static.power.law.game* function in the *igraph* package with the argument *finite.size.correction = TRUE*. In this study, we avoided the emergence of self-loops and multiple edges. *γ* was set to 2.2 because *γ* in many real-world networks is between 2 and 2.5 [38].

Following the work of Allesina and Tang [31], we considered five types of interaction matrices: random, mutualistic, competitive, predator–prey (parasitic), and a mixture of competition and mutualism interaction matrices. Following simulation-based studies using GLV equations [39–41], the (absolute) weights of interactions (i.e., the elements in interaction matrices *M*_*ij*_) were drawn from uniform distributions.

In the random interaction matrices, *M*_*ij*_ was drawn from a uniform distribution of [−*s*_max_, *s*_max_] if *A*_*ij*_ = 1, and *M*_*ij*_ = 0 otherwise, where *s*_max_ is the upper (lower) limit for interaction strength. Given the definitions of mutualistic, competitive, and predator–prey (parasitic) interactions (see below for details), the random interaction matrices generated contain a mixture of these interaction types. For large *n*, in particular, mutualistic, competitive, and predator–prey interactions occur in the ratio of 1:1:2.

A mutualistic interaction between species *i* and *j* indicates that *M*_*ij*_ > 0 and *M*_*ji*_ > 0 because the species positively affect each other’s growth. In mutualistic interaction matrices, *M*_*ij*_ was drawn from a uniform distribution of (0, *s*_max_] if *A*_*ij*_ = 1, and *M*_*ij*_ = 0 otherwise. It should be noted that *M*_*ji*_ is also positive if *A*_*ij*_ = 1 because *A*_*ij*_ = *A*_*ji*_, but *A*_*ij*_ is independent from *M*_*ij*_.

A competitive interaction between species *i* and *j* indicates that *M*_*ij*_ < 0 and *M*_*ji*_ < 0 because the species negatively affect each other’s growth. In competitive interaction matrices, *M*_*ij*_ was drawn from a uniform distribution of [−*s*_max_, 0) if *A*_*ij*_ = 1, and *M*_*ij*_ = 0 otherwise. It should be noted that *M*_*ji*_ is also negative if *A*_*ij*_ = 1 because *A*_*ij*_ = *A*_*ji*_, but *A*_*ij*_ is independent from *M*_*ij*_.

Following a previous study [31], we generated interaction matrices consisting of a mixture of mutualistic and competitive interactions. For each species pair (*i*, *j*)_*i* < *j*_, we obtained a random value *p*_1_ from a uniform distribution of [0, 1] if *A*_*ij*_ = 1. After, *M*_*ij*_ and *M*_*ji*_ were independently drawn from a uniform distribution of (0, *s*_max_] if *p*_1_ ≤ *p*_*C*_ from a uniform distribution of [−*s*_max_, 0) otherwise where *p*_*C*_ corresponds to the ratio of competitive interactions to all interactions. It should be noted that *M*_*ij*_ = 0 if *A*_*ij*_ = 0.

A predator–prey (parasitic) interaction between species *i* and *j* indicates that *M*_*ij*_ and *M*_*ji*_ have opposite signs (e.g., whenever *M*_*ij*_ > 0, then *M*_*ji*_ < 0) because species *i* (*j*) positively contributes to the growth of species *j* (*i*), but the growth of species *i* (*j*) is negatively affected by species *j* (*i*). The predator–prey interaction matrices were generated as follows: for each species pair (*i*, *j*)_*i* < *j*_, we obtained a random value *p*_2_ from a uniform distribution of [0, 1] if *A*_*ij*_ = 1. If *p*_2_ ≤ 0.5, *M*_*ij*_ was drawn from a uniform distribution of [−*s*_max_, 0) and *M*_*ji*_ was drawn from a uniform distribution of (0, *s*_max_], while if *p*_2_ > 0.5 we did the opposite: *M*_*ij*_ and *M*_*ji*_ were independently drawn from uniform distributions (0, *s*_max_] and [−*s*_max_, 0), respectively. It should be noted that *M*_*ij*_ = 0 if *A*_*ij*_ = 0.

To investigate the effect of predator–prey interactions on co-occurrence network performance, we also considered interaction matrices consisting of a mixture of competitive and predator–prey interactions. For each species pair (*i*, *j*)_*i* < *j*_, we obtained a random value *p*_3_ from a uniform distribution of [0, 1] if *A*_*ij*_ = 1; then, *M*_*ij*_ and *M*_*ji*_ were determined based on to the above definition of competitive interactions if *p*_3_ ≤ *p*_*C*_, otherwise they were determined based on the above definition of predator–prey interactions. It should be noted that *M*_*ij*_ = 0 if *A*_*ij*_ = 0.

To obtain species abundances using the *n*-species GLV equations, we used the *generateDataSet* function in the R package *seqtime* (version 0.1.1) [40]; environmental perturbance was excluded for simplicity. Following Faust et al. [40], the GLV equations were numerically solved with initial species abundances that were independently drawn from a Poisson distribution with mean of 100 (i.e., the total number of individuals is 100*n*). Following previous studies [40, 41], the growth rates of species (*r*_*i*_) were independently drawn from a uniform distribution of (0,1]. Following the default options of the *generateDataSet* function, species abundances were obtained at the 1000-time step. We empirically confirmed that species abundances reached a steady state before the 1000-time step (Additional file 1: Figure S1). The absolute abundances were converted into relative values. The relative abundance *P*_*i*_ of species *i* was calculated as ${N}_i/{\sum}_{j=1}^n{N}_j$ where *N*_*i*_ is the absolute abundance of species *i* at the time step. The resulting absolute and relative abundances were recorded. This process was repeated until the desired number of samples was obtained. The source codes for dataset generation are available in Additional file 2.

### Co-occurrence network methods

We evaluated the extent to which the nine co-occurrence network methods decipher original interaction patterns (i.e., adjacency matrix *A*_*ij*_) from the generated relative abundance (compositional) dataset based on associations between species abundances (see Additional file 1: Figure S2). In particular, six correlation-based methods were investigated: Pearson’s correlation (PEA), Spearman’s correlation (SPE), MIC [14], SparCC [16], REBACCA [17], and CCLasso [18]. Moreover, three graphical model-based methods were also investigated: Pearson’s partial correlation (PPEA), Spearman’s partial correlation (PSPE), and SPIEC-EASI [20].

The pair-wise Pearson’s and Spearman’s correlation matrices were calculated using the *cor* function in R with the arguments *method = “pearson”* and *method = “spearman”*, respectively. The pair-wise MICs were determined using the *mine* function in the R package *minerva* (version 1.5). We also estimated the ecological microbial networks using the SparCC, REBACCA, and CCLasso algorithms. The SparCC program was downloaded from bitbucket.org/yonatanf/sparcc on November 11, 2018, and it ran under the Python environment (version 2.7.15; www.python.org). The REBACCA program was obtained from faculty.wcas.northwestern.edu/~hji403/REBACCA.htm on November 16, 2018. The CCLasso program was obtained from github.com/huayingfang/CCLasso on November 13, 2018. REBACCA and CCLasso ran under the R environment. We used SparCC, REBACCA, and CCLasso with the default options, but we provided the option *pseudo = 1* when using CCLasso for convergence.

The Pearson’s and Spearman’s partial correlation coefficients were calculated using the *pcor* function in the R package *ppcor* (version 1.1) with the arguments *method = “pearson”* and *method = “spearman”*, respectively. We also obtained the co-occurrence networks using the SPIEC-EASI algorithm with neighborhood selection. The SPIEC-EASI program was downloaded from github.com/zdk123/SpiecEasi on November 13, 2018. We used SPIEC-EASI in the R environment with the default options.

### Evaluating co-occurrence network performance

Following previous studies [20], to evaluate co-occurrence network performance (i.e., how well the estimated co-occurrence network describes the original interaction pattern *A*_*ij*_), we obtained the precision–recall (PR) curve based on confidence scores of interactions for each inference result, comparing the lower triangular parts of confidence score matrices and *A*_*ij*_ because the matrices were symmetric. It should be noted that the lower triangular parts were vectorized after excluding the diagonal terms. The precision and recall were calculated by binarizing the confidence scores at a threshold. The PR curve was obtained as the relationship between precision and recall for different threshold. We used the absolute correlation coefficients for the Pearson’s correlation, Spearman’s correlation, MIC, Pearson’s partial correlation, Spearman’s partial correlation, SparCC, and CCLasso for the confidence scores. Following previous studies [17, 20], edge-wise stability scores were used for REBACCA and SPIEC-EASI. Furthermore, we summarized the PR curve with the area under the PR curve (AUPR). The AUPR values were averaged over 50 iterations of dataset generation and performance evaluation with randomly assigned parameters for each iteration. The PR curves and AUPR values were obtained using the *pr.curve* function in the R package *PRROC* (version 1.3.1). We also computed the baseline-corrected AUPR values because positive and negative ratios affect PR curves. The baseline-corrected AUPR value was defined as (AUPR_obs_ – AUPR_rand_) / (1 – AUPR_rand_), where AUPR_obs_ and AUPR_rand_ correspond to the observed AUPR value and the AUPR value obtained from random prediction (i.e., 2*m*/[*n*(*n* − 1)] = 〈*k*〉/(*n* − 1)), respectively. The source codes for evaluating co-occurrence network performance are available in Additional file 2.

It is important to mention that the problem of false-negative interactions may occur when we do performance analysis based on adjacency matrices *A*_*ij*_: negligible interactions (i.e., when both |*M*_*ij*_*|* and |*M*_*ji*_| have very small values) have negligible effects on population dynamics and act as no interaction. It may happen even if the corresponding nodes are connected (i.e., *A*_*ij*_ = *A*_*ji*_ = 1). However, this problem hardly affects co-occurrence network performance. Supposing such false-negative interactions occur if |*M*_*ij*_| < *s*_*c*_ and |*M*_*ji*_| < *s*_*c*_ when *A*_*ij*_ = *A*_*ji*_ = 1 where *s*_*c*_ is a small value, the expected ratio of false-negative interactions to all interacting pairs (edges) is described as (*s*_*c*_ / *s*_max_)^2^ because |*M*_*ij*_| and |*M*_*ji*_| are independently drawn from the uniform distribution of (0, *s*_max_]. Assuming that *s*_max_ = 0.5 and *s*_*c*_ = 0.01, for example, 0.04% of *m* edges indicate false-negative interactions.

## Results

### Compositional-data co-occurrence network methods performance did not exceed that of classical methods

We generated relative abundance datasets through population dynamics. In particular, we used the GLV equations with an interaction matrix *M*_*ij*_ constructed from an interaction pattern *A*_*ij*_ (random, small-world, or scale-free network structure) by considering types of interaction matrices (random, mutualistic, competitive, predator–prey (parasitic), or mixture of competition and mutualism interaction matrices). We investigated how well co-occurrence network methods decipher interaction patterns from relative abundance data by evaluating the consistency between the confidence score matrices obtained from the methods and *A*_*ij*_ based on the (baseline-corrected) AUPR values.

**Fig. 1:**
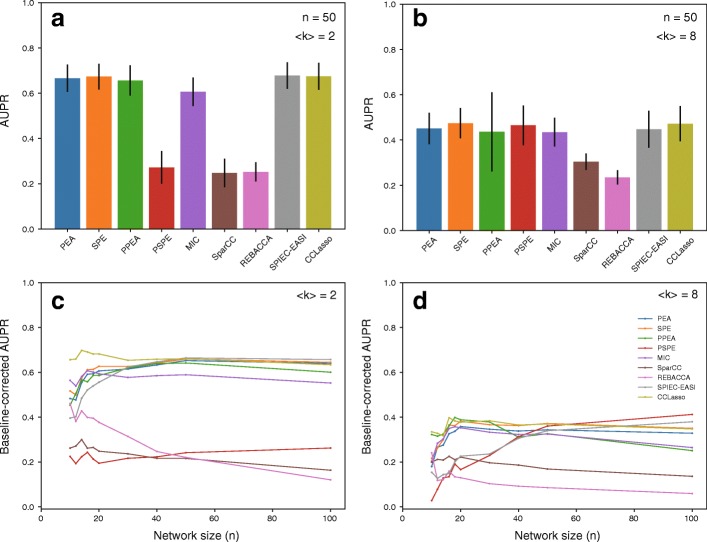
Differences in the co-occurrence network performance between methods. AUPR values for 50-node networks with average degree 〈*k*〉 = 2 (**a**) and 〈*k*〉 = 8 (**b**). Error bars indicate standard deviations. Relationships between the baseline-corrected AUPR value and network size *n* for 〈*k*〉 = 2 (**c**) and 〈*k*〉 = 8 (**d**). Random interaction matrices and random network structure were considered. *s*_max_ was set to 0.5. The number of samples was set to 300

We investigated the case of random interaction matrices constructed based on random network structures (Fig. 1). We found that co-occurrence network performance (AUPR value) was moderate. For example, the AUPR value was at most ~ 0.65 when network size (the number of species) *n* = 50 and average degree 〈*k*〉 = 2 (Fig. 1a), and it was at most ~ 0.45 when *n* = 50 and 〈*k*〉 = 8 (Fig. 1b). As expected from limitations due to the constant sum constraint, the performance of the classical co-occurrence network methods (e.g., Pearson’s correlation) generally decreased when using compositional data (Additional file 1: Figure S3), and the performance of the partial correlation-based methods declined largely.

More importantly, we found that the performance of the compositional-data co-occurrence network methods were almost equal to or less than that of classical methods, excluding Spearman’s partial correlation-based method; in particular, the performance of some compositional-data methods was lower than that of the classical methods. Specifically, the AUPR values of SparCC, an earlier compositional-data method, were lower than those of Pearson’s correlation [*p* < 2.2e–16 using *t*-test when *n* = 50 and 〈*k*〉 = 2 (Fig. 1a) and *p* < 2.2e–16 using *t*-test when *n* = 50 and 〈*k*〉 = 8 (Fig. 1b)]. Moreover, The AUPR values of REBACCA, a later compositional-data method, were also lower than those of Pearson’s correlation [*p* < 2.2e–16 using *t*-test when *n* = 50 and 〈*k*〉 = 2 (Fig. 1a) and *p* < 2.2e–16 using *t*-test when *n* = 50 and 〈*k*〉 = 8 (Fig. 1b)]. For 50-node networks, the performance of CCLasso and SPIEC-EASI was similar to that of classical methods when 〈*k*〉 = 2 (Fig. 1a) and 〈*k*〉 = 8 (Fig. 1b). However, the performance of later compositional-data methods (e.g., CCLasso) was higher than that of the earlier compositional-data method (i.e., SparCC). Specifically, the AUPR values of CCLasso were lower than those of SparCC [*p* < 2.2e–16 using *t*-test when *n* = 50 and 〈*k*〉 = 2 (Fig. 1a) and *p* = 3.2e–7 using *t*-test when *n* = 50 and 〈*k*〉 = 8 (Fig. 1b)].

The graphical model-based methods were not more efficient than the correlation-based methods. Spearman’s partial correlation-based method was inferior to Pearson’s correlation-based method (*p* < 2.2e–16 using *t*-test) and Spearman’s correlation-based method (*p* < 2.2e–16 using *t*-test) when *n* = 50 and 〈*k*〉 = 2 (Fig. 1a); however, the AUPR value of Spearman’s partial correlation-based method was similar to that of Pearson’s and Spearman’s correlation-based methods when *n* = 50 and 〈*k*〉 = 8 (Fig. 1b). Both Pearson’s partial correlation-based method and Pearson’s correlation-based method exhibited similar performance. The performance of the graphical model-based method for compositional data (SPIEC-EASI) was similar to that of other correlation-based methods (e.g., Pearson’s correlation), although it was higher than that of the correlation-based methods for compositional data. Specifically, the AUPR values of SPIEC-EASI were higher than those of SparCC [*p* < 2.2e–16 using *t*-test when *n* = 50 and 〈*k*〉 = 2 (Fig. 1a) and *p* < 2.2e–16 using *t*-test when *n* = 50 and 〈*k*〉 = 8 (Fig. 1b)].

Co-occurrence network performance was evaluated when the average degree (Fig. 1a and b) and number of nodes (network size; Fig. 1c and d) varied; moreover, it was also examined for other types of network structure: small-world networks (Additional file 1: Figure S4) and scale-free networks (Additional file 1: Figure S5).

### Interaction patterns in more complex networks are harder to predict

It is noteworthy that network size, average degree, and network type affected co-occurrence network performance. The co-occurrence network performance (baseline-corrected AUPR values) varied with network size in some methods (Fig. 1c and d). In particular, the performance of Spearman’s partial correlation-based method increased with network size in dense networks, while the performance of REBACCA decreased with network size in sparse networks. However, co-occurrence network performance was nearly independent of network size when *n* > 20 in most methods. The interaction patterns in small networks were poorly predicted; the co-occurrence network methods are not suitable for capturing interaction patterns in small networks. The differences in the performance between the co-occurrence network methods and random predictions were not remarkable because the degree of freedom was low in small networks.

**Fig. 2:**
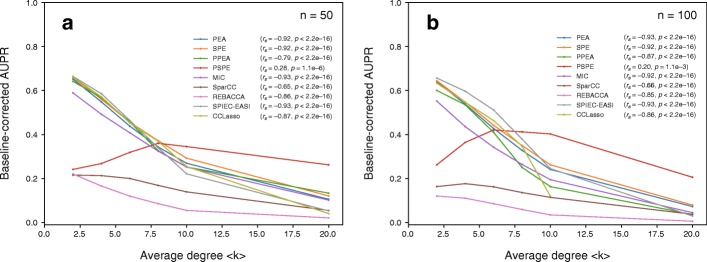
Relationships between co-occurrence network performance (baseline-corrected AUPR value) and average degree when network size *n* = 50 (**a**) and *n* = 100 (**b**). Random interaction matrices and random network structure were considered. *s*_max_ was set to 0.5. The number of samples was set to 300. The baseline-corrected AUPR values of CCLasso were not calculated when 〈*k*〉 > 10 in 100-node networks because of high computational costs. *r*_*s*_ and *p* indicate the Spearman’s rank correlation coefficient and the associated *p*-value. The raw values (i.e., the values before averaging) were used for calculating *r*_*s*_

More importantly, the interaction patterns in denser networks generally were more difficult to predict; in particular, we observed general negative correlations between the performance (baseline-corrected AUPR value) and average degree when *n* = 50 (Fig. 2a) and *n* = 100 (Fig. 2b). However, the performance of Spearman’s partial correlation-based method (PSPE) increased for 〈*k*〉 < ~8 and decreased for 〈*k*〉 ≥ ~8 when *n* = 50 and 100. This method exhibited the highest performance for dense networks while it exhibited relatively low performance for sparse networks; nonetheless, it should be noted that this method poorly predicted interactions patterns (the baseline-corrected AUPR value was at most ~ 0.4 when 〈*k*〉 ≥ ~8). The co-occurrence network performance slightly increased when using more samples (Additional file 1: Figure S6); in particular, we investigated cases in which network size (*n* = 50 and 100) and average degree (〈*k*〉 = 2 and 8) differed and found that co-occurrence network performance was almost independent of sample number when it exceeds 200 in most methods.

**Fig. 3:**
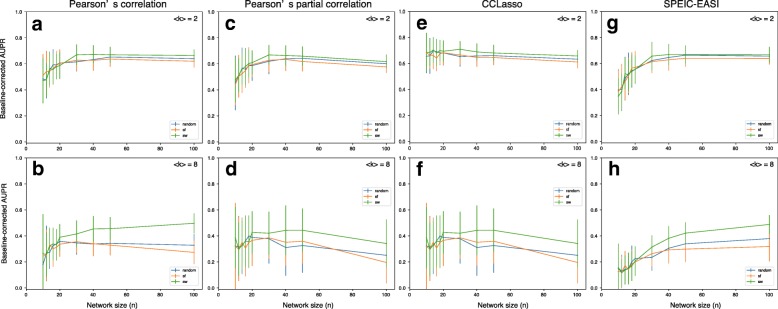
Relationships between co-occurrence network performance (AUPR value) and network size *n* according to the network types: random networks (random), scale-free networks (sf), and small-world networks (sw). Random interaction matrices were considered. The cases of sparse networks (〈*k*〉 = 2; top panels) and dense networks (〈*k*〉 = 8; bottom panels) are shown. As representative examples, Pearson’s correlation-based method (a classical correlation-based method; **a** and **b**), Pearson’s partial correlation-based method (a classical graphical model-based method; **c** and **d**), CCLasso (a correlation-based method for compositional data; **e** and **f**), and SPEIC-EASI (a graphical model-based method for compositional data; **g** and **h**) are shown. *s*_max_ was set to 0.5. The number of samples was set to 300

The correlations between the baseline-corrected AUPR values and average degree were also investigated in small-world networks (Additional file 1: Figure S4 and S7) and scale-free networks (Additional file 1: Figures S5 and S8), and the negative correlations between the baseline-corrected AUPR values and average degree were specifically observed. However, co-occurrence network performance moderately varied according to network type in large and dense networks when focusing on each inference method (Fig. 3). In particular, we investigated Pearson’s correlation-based method (a classical correlation-based method; Fig. 3a and b), Pearson’s partial correlation-based method (a classical graphical model-based method; Fig. 3c and d), CCLasso (a correlation-based method for compositional data; Fig. 3e and f), and SPEIC-EASI (a graphical model-based method for compositional data; Fig. 3g and h). In general, the lowest performance was observed for scale-free networks, while the highest performance was observed for small-world networks (Fig. 3). Specifically, the baseline-corrected AUPR values for scale-free networks were lower than those for small world networks when *n* = 100 and 〈*k*〉 = 8 (*p* < 2.2e–16 using *t*-test for Pearson’s correlation-based method; *p* = 7.7e–5 using *t*-test for Pearson’s partial correlation-based method; *p* = 0.027 using *t*-test for CCLasso; *p* = 1.9e–13 using *t*-test for SPEIC-EASI). Moreover, the baseline-corrected AUPR values for scale-free networks were lower than those for random networks when *n* = 100 and 〈*k*〉 = 8 for Pearson’s correlation-based method (*p* = 2.9e–3 using *t*-test) and SPEIC-EASI (*p* = 7.4e–3 using *t*-test).

The results indicating that compositional-data co-occurrence network methods were not more efficient than classical methods and that interaction patterns in more complex networks are more difficult to predict (Figs. 1, 2 and 3) were also generally confirmed in the other types of interactions matrices: competitive (Additional file 1: Figures S9–S11), mutualistic (Additional file 1: Figures S12 and S13), predator–prey (Additional file 1: Figures S14–S16), and mutualism-competition mixture interaction matrices (Additional file 1: Figures S17–S19).

### Predator-prey (parasitic) interactions decrease co-occurrence network performance

**Fig. 4:**
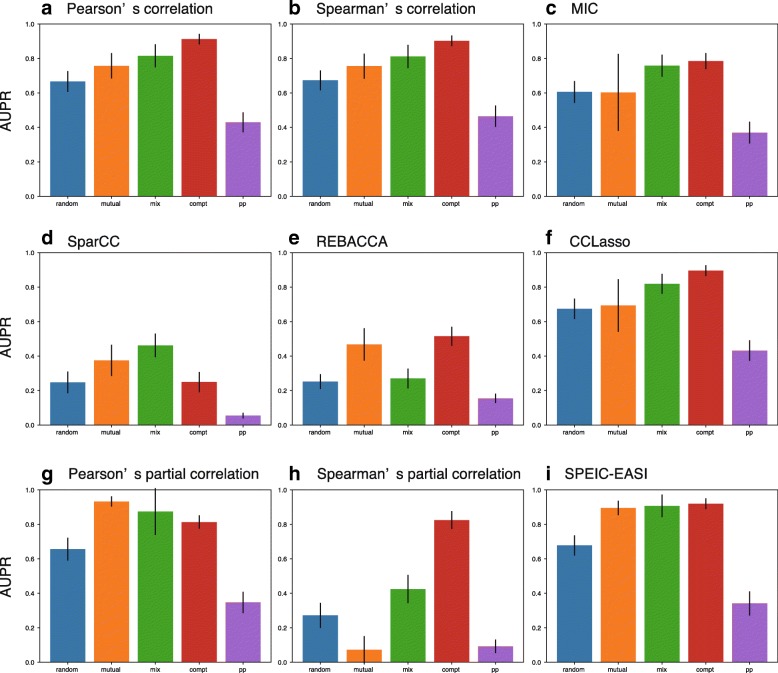
Effects of the community type on co-occurrence network performance (AUPR value) in the cases of Pearson’s correlation-based method (**a**), Spearman’s correlation-based method (**b**), MIC-based method (**c**), SparCC (**d**), REBACCA(**e**), CCLasso (**f**), Pearson’s partial correlation-based method (**g**), Spearman’s partial correlation-based method (**h**), and SPEIC-EASI (**i**). Vertical-axis labels correspond to the community types: random community (random; blue), mutualistic community (mutual; orange), competition–mutualism mixture community (mix; green), competitive community (compt; red), and predator–prey (parasitic) community (pp; purple). Network size *n* = 50 and average degree 〈*k*〉 = 2. Random network structure was considered. *s*_max_ was set to 0.5. The number of samples was set to 300. Error bars indicate standard deviations

The types of interaction matrices notably affected co-occurrence network performance (Fig. 4). Specifically, in most methods, the interaction patterns in predator–prey (parasitic) communities (interaction matrices) were the most difficult to predict, while those in competitive communities were the easiest to predict. Specifically, the AUPR values for predator–prey communities were significantly lower than those for competitive communities for Pearson’s correlation-based method (*p* < 2.2e–16 using *t*-test; Fig. 4a), Spearman’s correlation-based method (*p* < 2.2e–16 using *t*-test; Fig. 4b), MIC-based method (*p* < 2.2e–16 using *t*-test; Fig. 4c), SparCC (*p* < 2.2e–16 using *t*-test; Fig. 4d), REBACCA (*p* < 2.2e–16 using *t*-test; Fig. 4e), CCLasso (*p* < 2.2e–16 using *t*-test; Fig. 4f), Pearson’s partial correlation-based method (*p* < 2.2e–16 using *t*-test; Fig. 4g), Spearman’s partial correlation-based method (*p* < 2.2e–16 using *t*-test; Fig. 4h), and SPEIC-EASI (*p* < 2.2e–16 using *t*-test; Fig. 4i). Additionally, co-occurrence network methods relatively accurately predicted interactions patterns in mutual communities and competition–mutualism mixture communities; however, they described the interaction patterns in random communities poorly. Specifically, the AUPR values for random communities also were significantly lower than those for competitive communities for Pearson’s correlation-based method (*p* < 2.2e–16 using *t*-test; Fig. 4a), Spearman’s correlation-based method (*p* < 2.2e–16 using *t*-test; Fig. 4b), MIC-based method (*p* < 2.2e–16 using *t*-test; Fig. 4c), REBACCA (*p* < 2.2e–16 using *t*-test; Fig. 4e), CCLasso (*p* < 2.2e–16 using *t*-test; Fig. 4f), Pearson’s partial correlation-based method (*p* < 2.2e–16 using *t*-test; Fig. 4g), Spearman’s partial correlation-based method (*p* < 2.2e–16 using *t*-test; Fig. 4h), and SPEIC-EASI (*p* < 2.2e–16 using *t*-test; Fig. 4i). Similar tendencies of the effect of interaction types on co-occurrence network performance were observed in varying network sizes (i.e., *n* = 20 and 100; Additional file 1: Figure S20), average degrees (i.e., 〈*k*〉 = 4 and 8; Additional file 1: Figure S21), and network structures (i.e., small-world and scale-free network structures; Additional file 1: Figure S22).

**Fig. 5:**
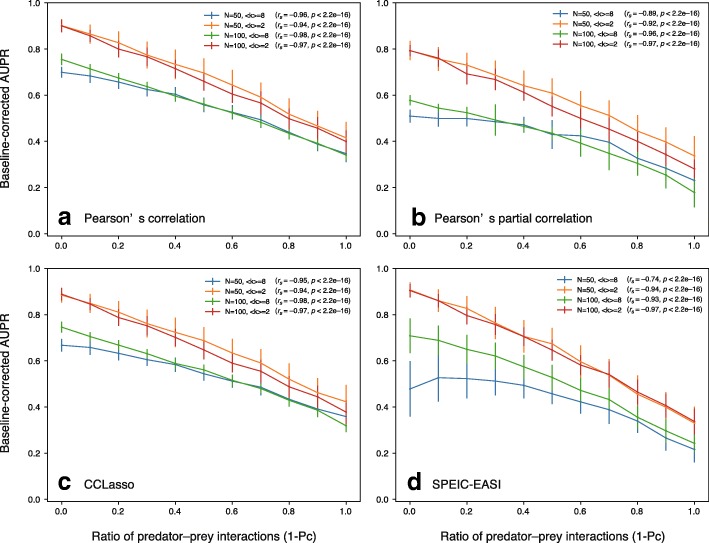
Relationship between co-occurrence network performance (baseline-corrected AUPR value) and the ratio of predator–prey (parasitic) interactions (1 – *p*_*C*_). As representative examples, Pearson’s correlation-based method (**a**), Pearson’s partial correlation method (**b**), CCLasso (**c**), and SPIEC-EASI (**d**) are shown. For each method, the following cases are shown: network size *n* = 50 and average degree 〈*k*〉 = 2; *n* = 100 and 〈*k*〉 = 2; *n* = 50 and 〈*k*〉 = 8; and *n* = 100 and 〈*k*〉 = 8. Random interaction matrices and random network structure were considered. *s*_max_ was set to 0.5. The number of samples was set to 300. *r*_*s*_ and *p* indicate the Spearman’s rank correlation coefficient and the associated *p*-value. The raw values (i.e., the values before averaging) were used for calculating *r*_*s*_

We hypothesized that co-occurrence network performance decreases as the ratio of predator–prey (parasitic) interactions increases because the worst performance and second worst performance were observed for predator–prey and random communities, respectively. Note that almost half of the interactions are spontaneously set to predator–prey interactions in random communities (see “Generation of relative abundance data using a dynamical model” section). To test this hypothesis, we considered interaction matrices consisting of a mixture of competitive and predator–prey interactions because co-occurrence network performance was best and worst in competitive and predator–prey (parasitic) communities, respectively. In particular, we considered competition–parasitism mixture communities with the ratio *p*_*C*_ of competitive interactions to all interactions and investigated the relationship between the ratio of predator–prey interactions (i.e., 1 − *p*_*C*_) and AUPR values. As representative examples, we investigated Pearson’s correlation-based method (a classical correlation-based method; Fig. 5a), Pearson’s partial correlation method (a classical graphical model-based method; Fig. 5b), CCLasso (a correlation-based method for compositional data; Fig. 5c), and SPIEC-EASI (a graphical model-based for compositional data; Fig. 5d). As expected, we found negative correlations between co-occurrence network performance (AUPR value) and the ratio of predator–prey interactions (Fig. 5). Such negative correlations were also observed in cases with different network sizes (*n* = 50 and 100) and average degrees (〈*k*〉 = 2 and 8).

## Discussion

Inspired by previous studies [30], we evaluated how well co-occurrence network methods ﻿recapitulate microbial ecological networks using a population dynamics model; co-occurrence network methods are often used for discussing species interactions although they only infer ecological associations. We compared wide-ranging methods using realistic simulations. Our results provide additional and complementary insights into co-occurrence network approaches in microbiome studies.

The results indicate that compositional-data methods, such as SparCC and SPIEC-EASI, are less useful in inferring microbial ecological networks than previously thought. As shown in Fig. 1, the performance (AUPR values) of the compositional-data methods was moderate; furthermore, these compositional-data methods were not more efficient than the classical methods, such as Pearson’s correlation-based method. This result is inconsistent with previous studies [17, 18, 20]. This discrepancy was mainly due to differences in co-occurrence network method validation between this and previous studies. Specifically, previous studies generated abundance data from a multivariable distribution with a given mean and covariance matrix and examined how accurately co-occurrence network methods describe the original covariance matrix structure. However, this study considered species abundances determined through population dynamics (GLV equations) and examined how accurately the methods reproduced interaction patterns in ecological communities [30].

Population dynamics may lead to more complex associations between species abundances than parametric statistical models due to the nonlinearity of GLV equations. In compositional data co-occurrence network methods, such complex associations were likely difficult to detect because they assumed linear relationships between species abundances. The performance of Spearman’s correlation-based and MIC-based methods was almost equal to or higher than those of compositional-data methods because they can consider nonlinear associations, although such classical methods did not consider the effects of the constant sum constraint in compositional data. However, Pearson’s correlation-based method also exhibited a similar or higher performance than that of the compositional-data methods (Fig. 1), although it assumes linear relationships between species abundances in addition to the constant sum constraint. This may be due to approximation in the compositional-data methods, which estimate covariance matrices of the underlying absolute abundances from relative abundances using iterative approximation approaches. Thus, compositional-data methods may fail to correctly estimate the covariance structure of absolute abundance. According to a previous study [18], such a limitation is present in SparCC. REBACCA is similarly limited because its formalism is comparable to SparCC, although sparse methods are different between SparCC and REBACCA; thus, the performance of SparCC and REBACCA may have been low for similar reasons. On the other hand, CCLasso avoids these limitations [18], performing better than SparCC and REBACCA. However, more improvements may be required for CCLasso. It performed similarly to Pearson’s correlation-based method, which exhibited a higher performance using absolute abundances (particularly in sparse networks; Additional file 1: Figure S3). This indicates that CCLasso did not sufficiently infer the covariance structure of absolute abundances.

The graphical model-based methods were not more efficient than the correlation-based methods, although they do not consistently detect indirect associations (Fig. 1). In particular, Pearson’s and Spearman’s partial correlation-based (classical graphical model-based) methods were not more useful for inferring interaction patterns in ecological communities than Pearson’s and Spearman’s correlation-based (classical correlation-based) methods, and Spearman’s partial correlation-based method predicted interaction patterns in ecological communities poorly. This may have occurred due to the effects of the constant sum constraint in compositional data; specifically, these classical graphical model-based methods exhibited high performance with absolute abundances (Additional file 1: Figure S3). The effects of the constant sum constraint in partial correlation-based may be more significant than those in correlation-based methods, and errors due to the constant sum constraint in pairwise correlations (zero th-order partial correlations) may be amplified when calculating higher-order partial correlations. Thus, classical graphical-based models may be less useful than classical correlation-based models. The graphical model-based method for compositional data SPIEC-EASI has a similar problem. Similar to other correlation-based methods for compositional data (e.g., SparCC), SPIEC-EASI estimates absolute abundances from relative abundances. The estimated absolute abundances are not entirely accurate, which may be amplified in partial correlation (or regression) coefficients because SPIEC-EASI calculates coefficients based on the estimated values with the errors as classical partial correlation-based methods. CCLasso considers such errors through a loss function. Thus, CCLasso exhibited performance similar to SPIEC-EASI, although it did not directly consider avoiding indirect associations.

Interaction patterns in dense networks were difficult to predict (Fig. 2). This is generally because more indirect associations are observed; however, this may be because the assumption of sparsity in addition to errors due to absolute abundance approximation from relative abundances for compositional-data methods. This assumption is based on observations that real-world networks are very large and sparse [42], and sparsity is achieved through Lasso in the compositional-data methods. However, these Lasso-based methods might have overlooked important associations due to shrinkage and selection; Lasso may pick only one or a few strongly correlated variable pairs and shrink the rest to 0, i.e., no association [43]. To avoid this limitation, for example, we may need to consider the elastic-net and relaxed Lasso to estimate ecological associations under the sparsity assumption.

Additionally, interaction patterns in heterogeneous networks were the most difficult to detect while those in small-world networks, which are homogenous, were the easiest (Fig. 3). This result indicates that heterogeneity in degree distribution diminishes the performance of co-occurrence networks. This is consistent with the results of a previous study [30] in which it was observed that networks suffer from local hot spots of spurious correlation (indirect association) in the neighborhood of hub species that engage in many interactions. We expected that the graphical-based co-occurrence network method SPIEC-EASI avoided this limitation; however, the performance of SPIEC-EASI was similar to that of the other methods, as mentioned above. This may be due to the nonlinearity of species abundances and errors resulting from absolute abundance approximation from relative abundances. SPIEC-EASI may need to be improved.

Co-occurrence network performance increased with more samples (Additional file 1: Figure S6). More than 200 samples were required until plateaued performance was obtained. However, experimental studies may be able to consider fewer samples, down to 30 samples or less. Co-occurrence network methods that exhibit high performance with small samples must be developed.

More importantly, we found that interaction types affect co-occurrence network performance (Fig. 4). A previous study [10] also investigated the effects of interaction types. However, it used time-series data generated from GLV equations and is limited to small-scale networks to avoid system complexity. The behavior of the Lotka–Volterra systems is less understood for systems larger than two species, and small variations in the interaction matrix lead to significantly different abundance patterns. To investigate large-scale networks, we used steady-state species abundances generated from GLV equations, inspired by a previous study [30]. The data generation method performed by Berry and Widder [30] and in this study differs from the other previous study [10] although both studies considered GLV equations. Generated datasets are considered as a collection of steady-state snapshots (i.e., cross-sectional data) rather than time-series (longitudinal) data. Moreover, it is reasonable that observed species abundances are considered as cross-sectional data rather than longitudinal data in many microbiome studies. Despite the importance of time-series microbiome analysis [40, 44], time resolutions are still low due to technical limitations. As a result, we found that predator–prey (parasitic) interactions decrease co-occurrence network performance (AUPR values; Fig. 5). This result indicates detecting predator–prey interactions is more difficult than detecting other types of interactions, such as competitive and mutualistic interactions. This may be due to the behavioral complexity of predator–prey systems. The dynamics of predator–prey systems are known to be more complex than those of the other types of systems, even if the systems consist of only two species; in particular, predator–prey systems tend to oscillate [31]. Complex nonlinear associations are observed between the resulting species abundances obtained from predator–prey communities; thus, co-occurrence network methods failed to predict interaction patterns in these communities. This limitation may be important because predator–prey (parasitic) interactions play important roles in microbe–microbe interactions and human–microbiome interactions [45]. To avoid this issue, a compositional-data version of co-occurrence network methods based on maximal information-based nonparametric exploration, such as MIC, must be developed as MIC can detect such complex nonlinear associations [14].

However, further careful examination may be required. For example, more realistic dynamical models must be considered. For simplicity, we used classical GLV equations, and the conclusions we reached are limited to the species abundances generated from this classical model. The GLV equations may not reflect real-world microbial ecosystems. Ideally, we should have compared the generated data with real-world data; however, such comparisons were impossible because of a lack of compiled real-world data. The data on species (relative) abundances are available in several databases (e.g., Human Microbiome Project [4] and Earth Microbiome Project [5]); however, the model parameters (growth rate *r*_*i*_ and interaction matrix *M*_*ij*_) can be adjusted to nearly fit real-world abundance data using optimization methods. Thus, *r*_*i*_ and *M*_*ij*_ in real-world microbial ecosystems are needed to evaluate the validity of the generated abundance data; these real-world data are poorly investigated. However, this limitation does pose a significant problem because the main result is the difficulty in inferring microbial ecological networks using co-occurrence network methods. Real-world ecosystems are likely more complex. For example, species abundances, growth rates, and interaction matrices are temporally changed due to environmental perturbations. In this case, inferring ecological associations and interactions may be more difficult. Thus, it is believed that the main result also holds in more realistic ecosystems.

To more accurately detect ecological associations and directly detect species–species interactions, however, alternative methods are also needed. For example, a method grounded in maximum entropy models of statistical physics has been proposed to differentiate direct and indirect associations [46]. The difficulty of interpreting species–species interactions from co-occurrence data has been pointed out in community ecology [47]. To overcome this difficulty, Markov networks (Markov random fields) have been used for inferring species–species interactions from co-occurrence data in community ecology [48]. Dynamics (time series)-based methods are also useful. For example, convergent cross mapping [49] may be useful. This method is based on nonlinear state-space reconstruction and can distinguish causality in complex systems from correlation. The sparse S-map method [50] is a data-oriented equation-free modeling approach for multispecies ecological dynamics whose interaction topology is unknown, and it generates a sparse interaction network from a multivariate ecological time series without presuming any mathematical formulation for the underlying microbial processes. Another method, proposed by Xiao et al. [51], is based on Jacobian (community) matrices and can infer network topology and inter-taxa interaction types without assuming any particular population dynamics model from steady-state abundance data. Randomly distributed embedding [52] is a model-free framework that achieves accurate future-state prediction based on short-term high-dimensional data. However, these methods are not applicable to compositional data and must be improved. Thus, we did not consider these methods in this study.

## Conclusions

Our findings indicate that co-occurrence network methods are not efficient in interpreting interspecies interactions in microbiome studies because these methods only infer ecological associations. However, these results do not diminish the importance of co-occurrence network approaches. Co-occurrence network approaches remain a challenging research topic in the post-genomic era due to the importance of human [4] and ecological microbiomes [5]. Our findings highlight the need for further careful investigation of the validity of these widely used methods and development of more suitable approaches for inferring microbial ecological networks.

## Additional files

Additional file 1: Supplementary figures. (PDF 3162 kb)

Additional file 2: R source codes for generating datasets and for evaluating co-occurrence network performance. (ZIP 5 kb)

## Data Availability

All data generated and analyzed during this study are included in this published article and its supplementary information files.

## Abbreviations

AUPR: area under the precision–recall curve
CCLasso: correlation inference for compositional data through Lasso
GLV: generalized Lotka–Volterra
Lasso: least absolute shrinkage and selection operator
MIC: maximal information coefficient
PEA: Pearson’s correlation
PPEA: Pearson’s partial correlation
PR: precision–recall
PSPE: Spearman’s partial correlation
REBACCA: regularized estimation of the basis covariance based on compositional data
SparCC: sparse correlations for compositional data
SPE: Spearman’s correlation
SPIEC-EASI: sparse inverse covariance estimation for ecological association inference
