# Supplementary material for: Difficulty in inferring microbial community structure based on co-occurrence network approaches
Source: BMC Bioinformatics. 2019 Jun 13;20:329. doi: 10.1186/s12859-019-2915-1 (PMC6567618; doi:10.1186/s12859-019-2915-1)
Supplement: Supplementary file 1 — Supplementary figures. (PDF 3162 kb) [file 12859_2019_2915_MOESM1_ESM.pdf]

Supplementary information for “Difficulty in inferring microbial community structure based on co-occurrence network approaches”

Hokuto Hirano and Kazuhiro Takemoto

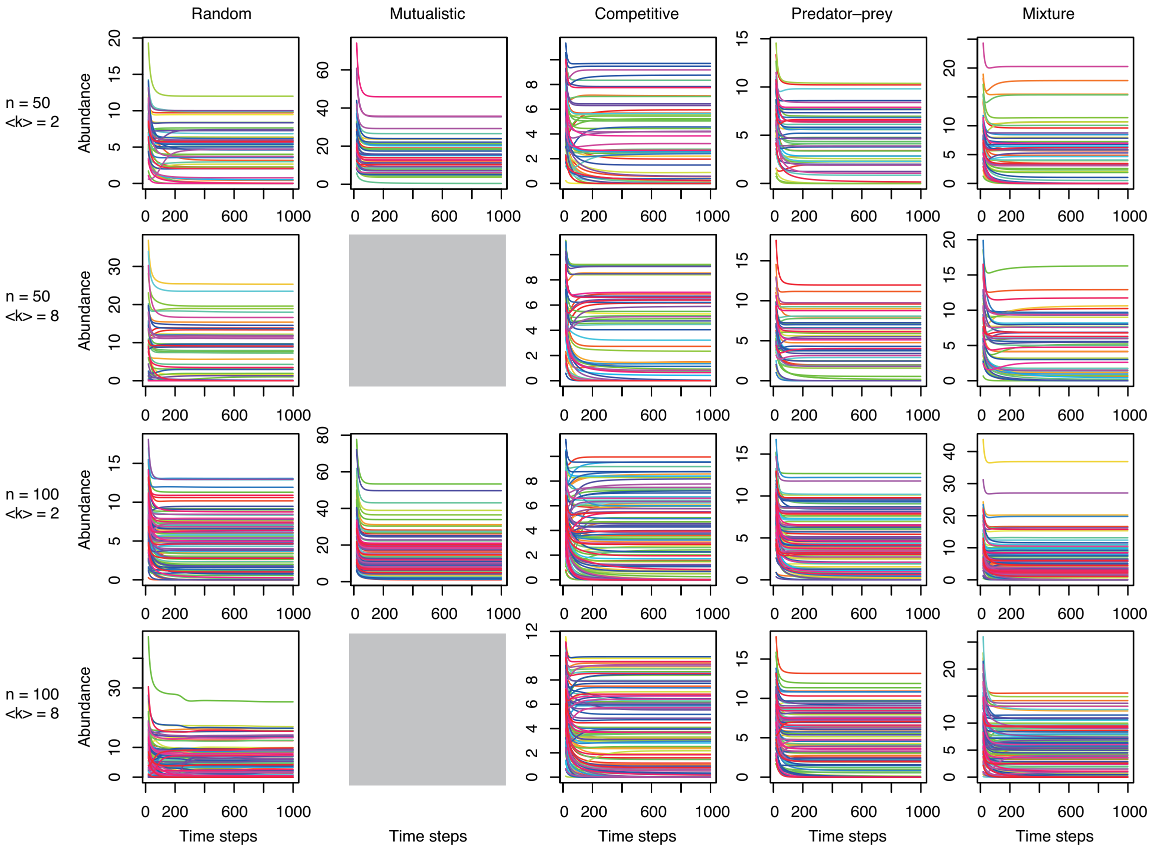

Figure S1: Examples of the dynamics of the GLV equations with random, mutualistic, competitive, predator–prey, and mixture of competition and mutualism interaction matrices. n and $\langle k \rangle$ correspond to network size (the number of species) and average degree, respectively. Random network structure was considered. $s_{\max}$ was set to 0.5. When $\langle k \rangle = 8$, mutualistic communities were not simulated due to numerical divergence in the simulation of the GLV equations. Tenfold abundances were displayed.

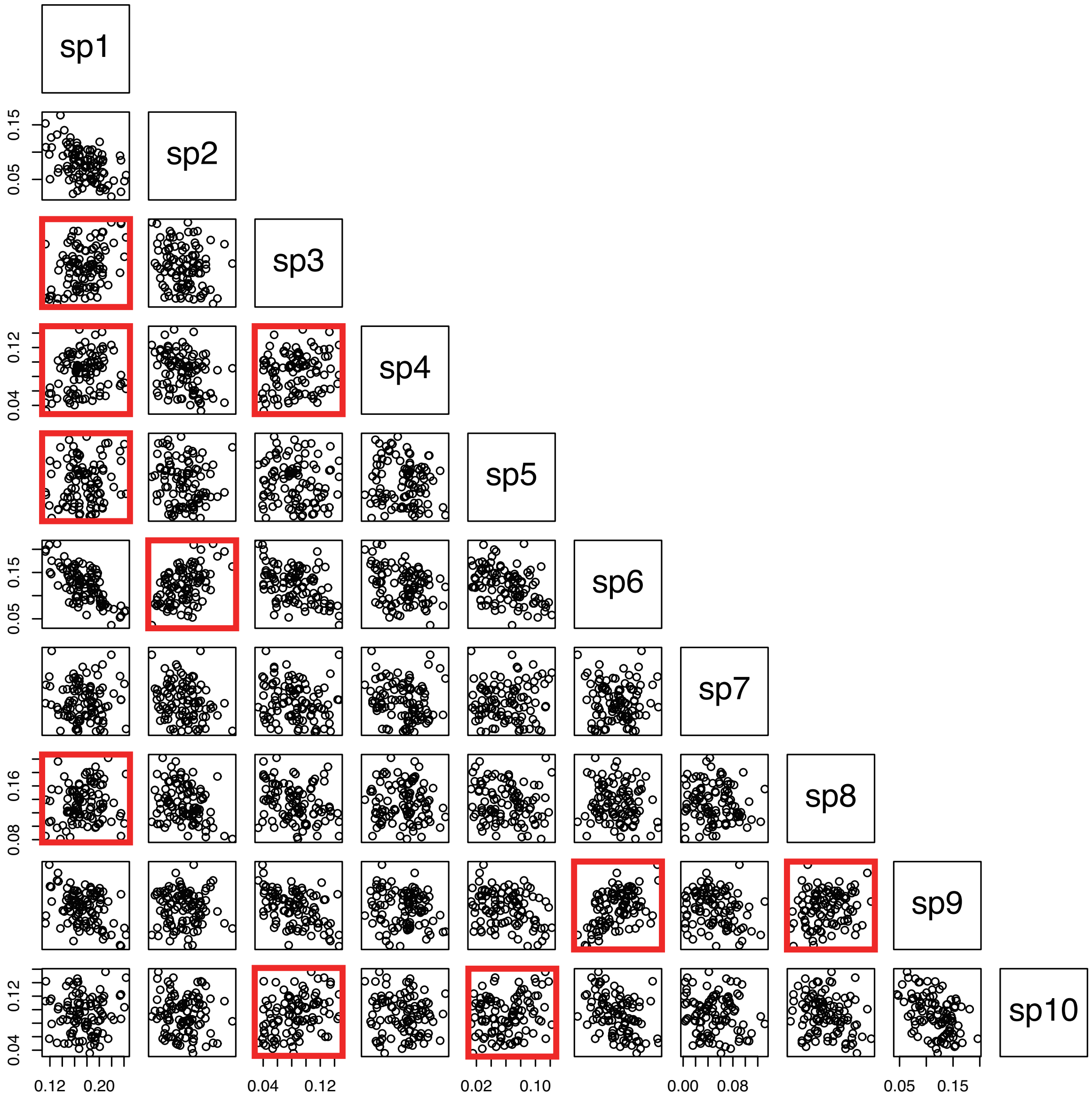

Figure S2: Example of correlations between species relative abundances generated from GLV equations. Mutualistic interaction matrices and random network structure with network size (the number of species) $n = 10$ and average degree $\langle k \rangle = 2$ were considered. $s_{\max}$ was set to 0.5. The number of samples was set to 100. sp$i$ indicates species i ($i = 1, \ldots, 10$). Red boxed panels indicate connected species pairs (i.e., $A_{ij} = 1$).

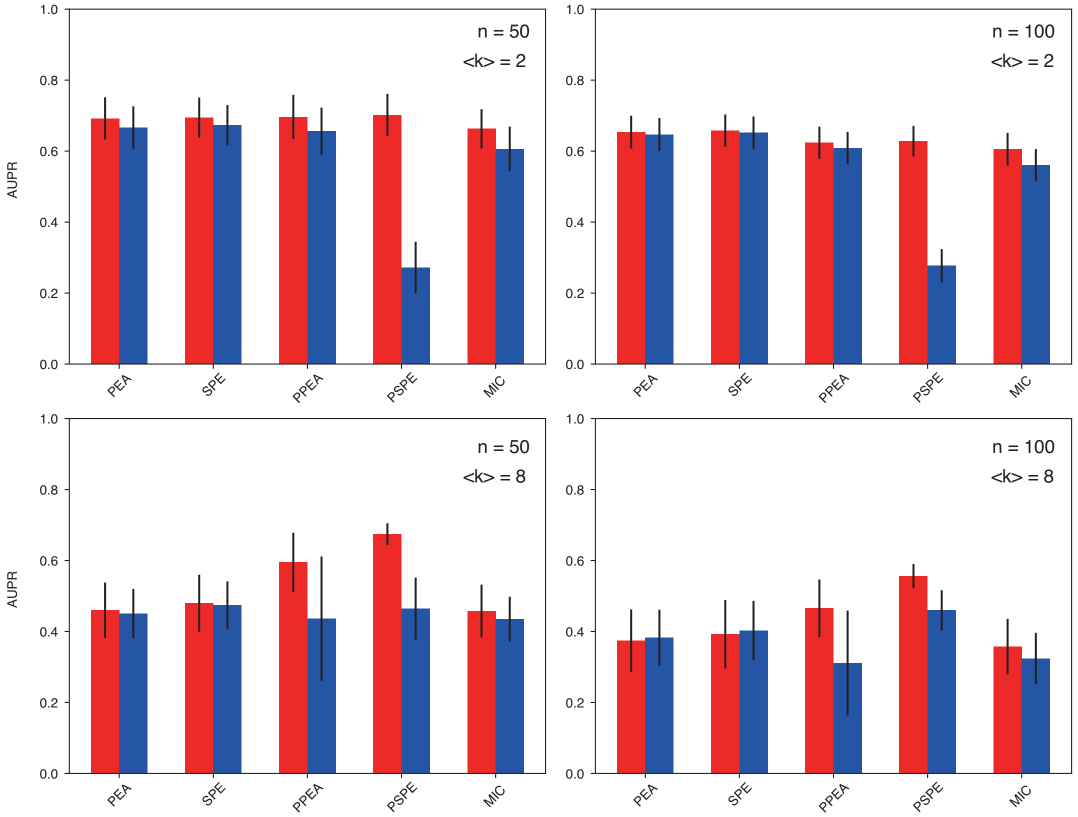

Figure S3: Comparison of the performance (AUPR value) of classical methods between using absolute abundances (red) and relative abundances (blue). Network size n = 50 (left panels) and n = 100 (right panels). Average degree $\langle k \rangle = 2$ (top panels) and $\langle k \rangle = 8$ (bottom panels). Random interaction matrices and random network structure were considered. $s_{\max}$ was set to 0.5. The number of samples was set to 300. Error bars indicate standard deviations.

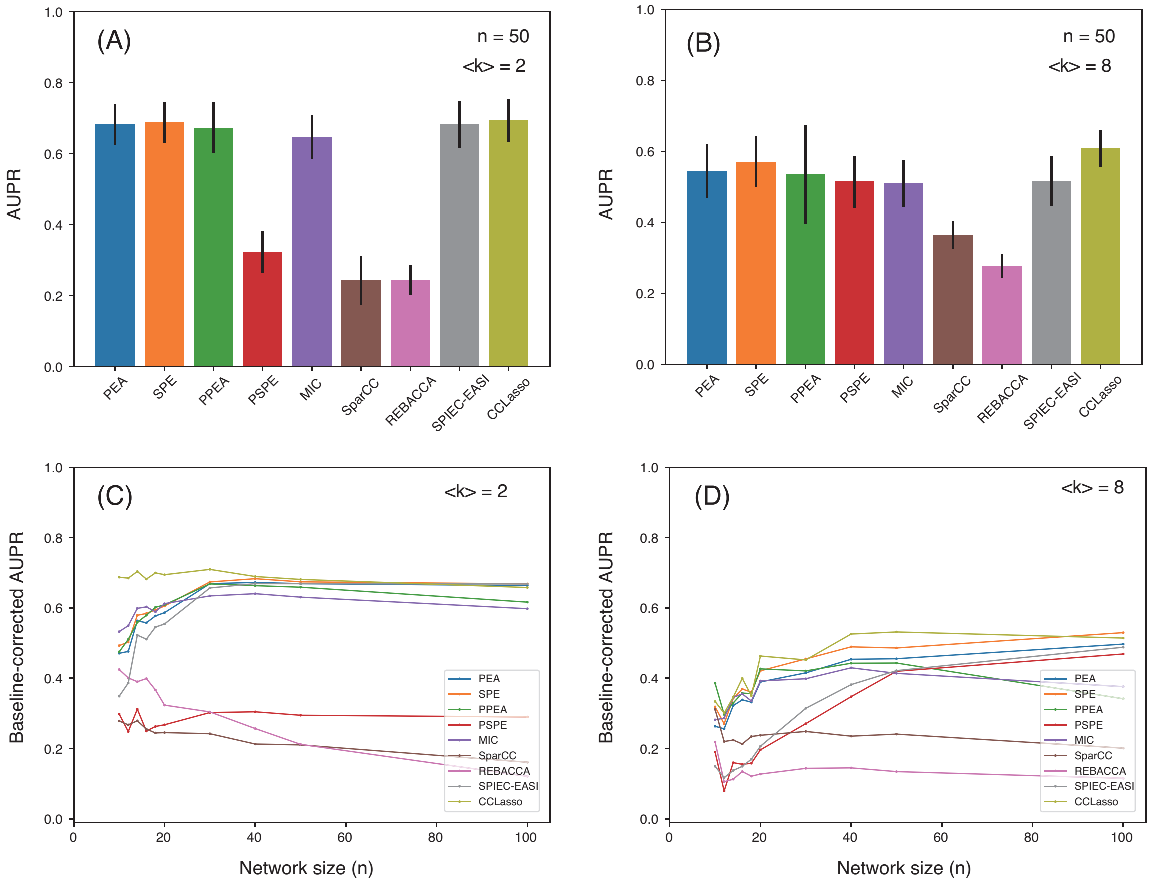

Figure S4: Differences in co-occurrence network performance between methods. AUPR values for 50-node networks with average degree $\langle k \rangle = 2$ (A) and $\langle k \rangle = 8$ (B) Error bars indicate standard deviations. Relationships between the baseline-corrected AUPR value and network size n for $\langle k \rangle = 2$ (C) and $\langle k \rangle = 8$ (D) Random interaction matrices and small-world network structure were considered. $s_{\max}$ was set to 0.5. The number of samples was set to 300.

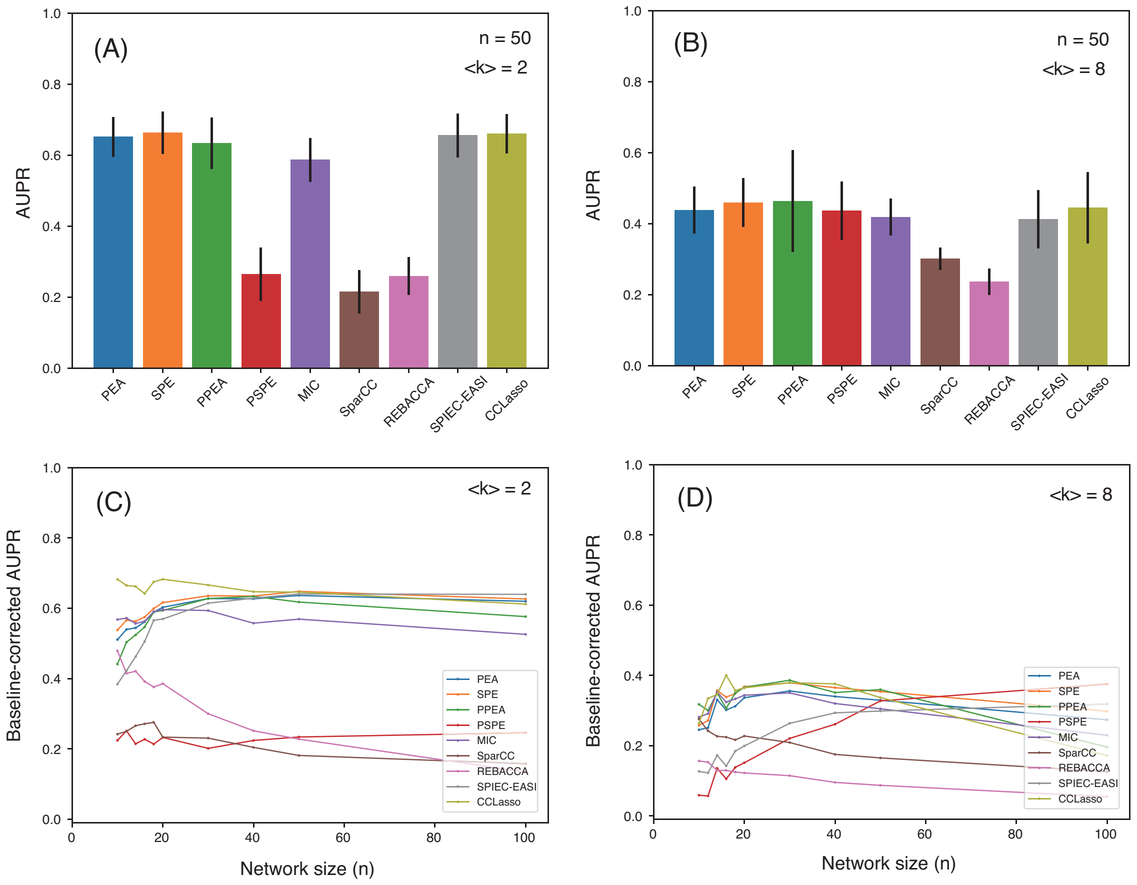

Figure S5: Differences in co-occurrence network performance between methods. AUPR values for 50-node networks with average degree $\langle k \rangle = 2$ (A) and $\langle k \rangle = 8$ (B) Error bars indicate standard deviations. Relationships between the baseline-corrected AUPR value and network size n for $\langle k \rangle = 2$ (C) and $\langle k \rangle = 8$ (D) Random interaction matrices and scale-free network structure were considered. $s_{\max}$ was set to 0.5. The number of samples was set to 300.

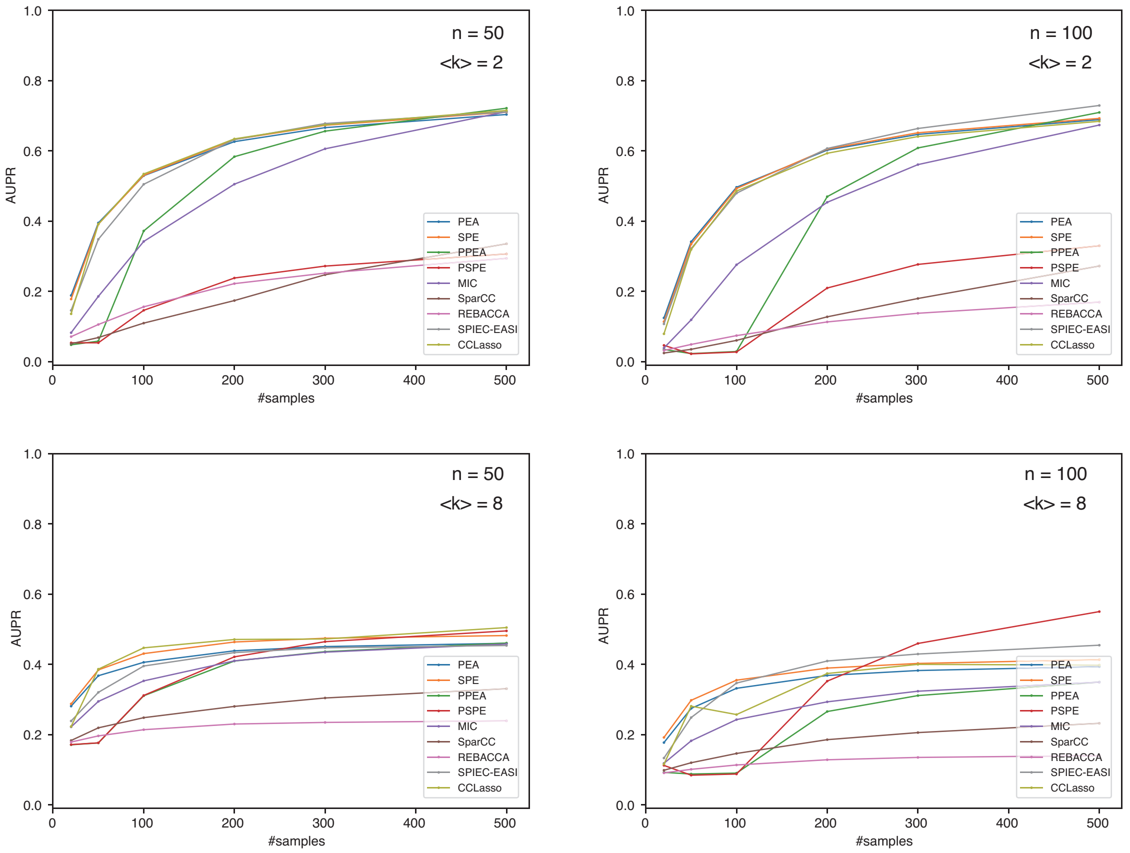

Figure S6: Relationships between co-occurrence network performance (AUPR value) and number of samples. Network size $n = 50$ (left panels) and $n = 100$ (right panels). Average degree $\langle k \rangle = 2$ (top panels) and $\langle k \rangle = 8$ (bottom panels). Random interaction matrices and random network structure were considered. $s_{\max}$ was set to 0.5. The number of samples was set to 300.

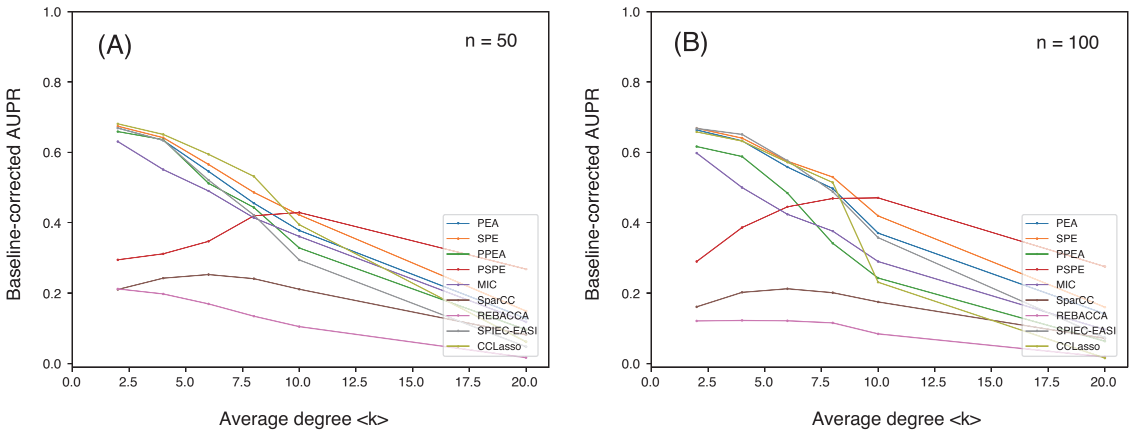

Figure S7: Relationships between co-occurrence network performance (baseline-corrected AUPR value) average degree when network size n = 50 (A) and n = 100 (B). Random interaction matrices and small-world network structure were considered. s_{max} was set to 0.5. The number of samples was set to 300.

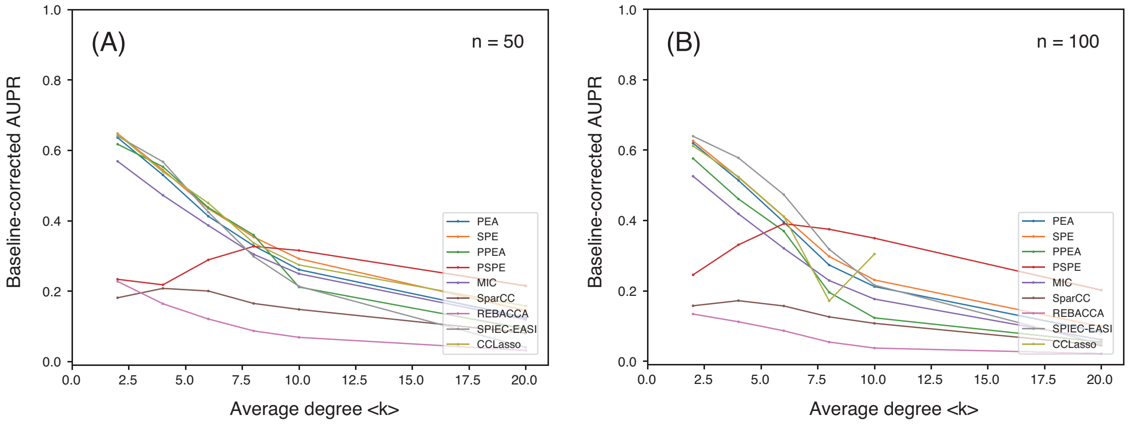

Figure S8: Relationships between co-occurrence network performance (baseline-corrected AUPR value) and average degree when network size n = 50 (A) and n = 100 (B). Random interaction matrices and scale-free network structure were considered. s_{max} was set to 0.5. The number of samples was set to 300. The baseline-corrected AUPR values of CCLasso were not calculated when $\langle k \rangle > 10$ in 100-node networks because of high computational costs.

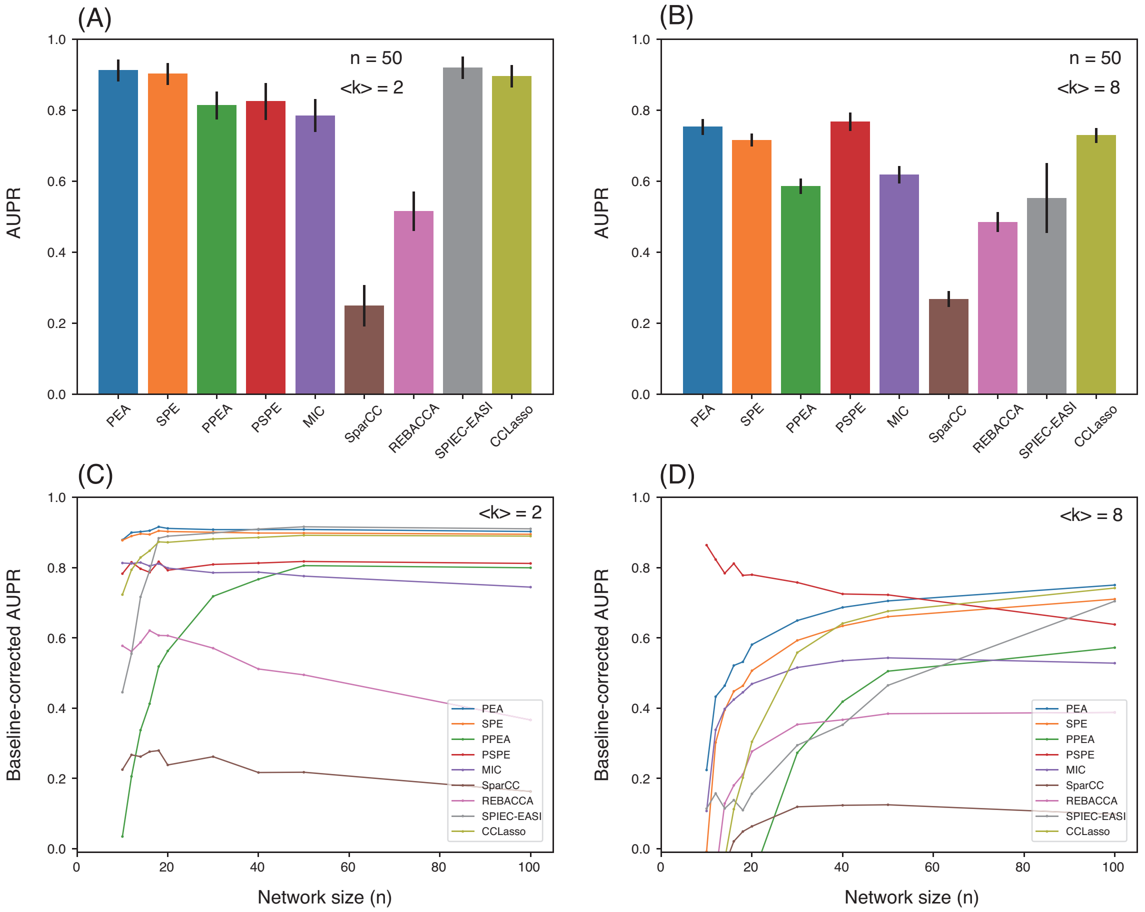

Figure S9: Differences in co-occurrence network performance between methods. AUPR values for 50-node networks with average degree $\langle k \rangle = 2$ (A) and $\langle k \rangle = 8$ (B). Error bars indicate standard deviations. Relationships between the baseline-corrected AUPR value and network size n for $\langle k \rangle = 2$ (C) and $\langle k \rangle = 8$ (D). Competitive interaction matrices and random network structure were considered. $s_{\max}$ was set to 0.5. The number of samples was set to 300.

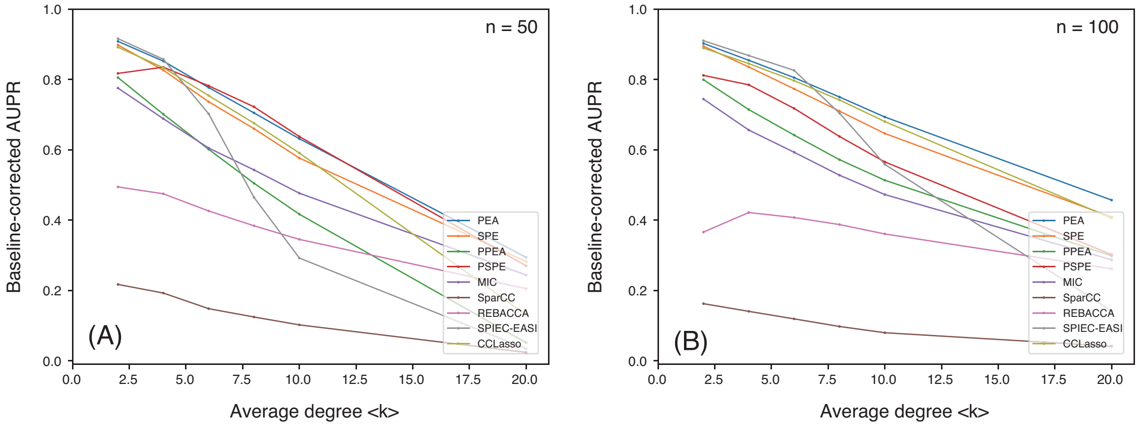

Figure S10: Relationships between co-occurrence network performance (baseline-corrected AUPR value) and average degree when network size n = 50 (A) and n = 100 (B). Competitive interaction matrices and random network structure were considered. s_{max} was set to 0.5. The number of samples was set to 300. The baseline-corrected AUPR values of CCLasso were not calculated when $\langle k \rangle > 10$ in 100-node networks because of high computational costs.

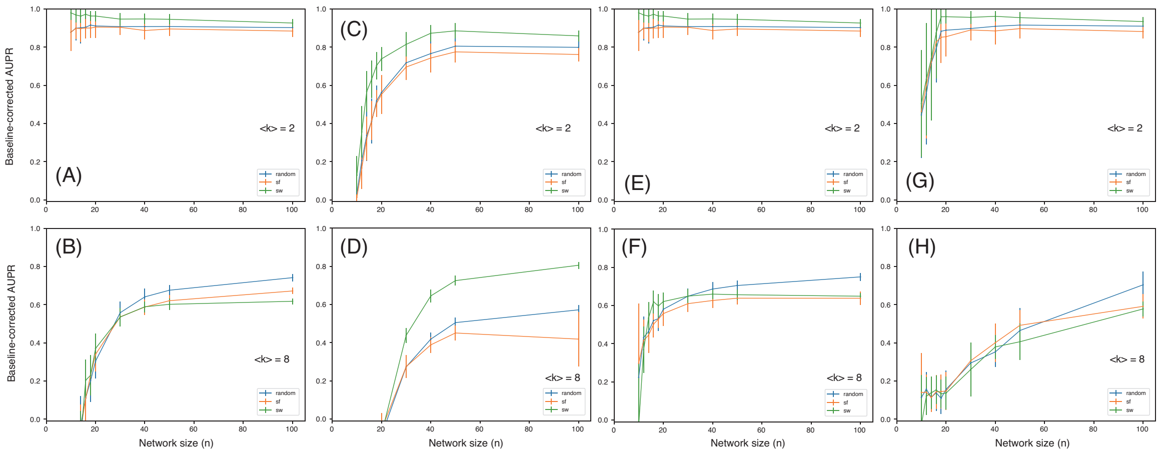

Figure S11: Relationships between co-occurrence network performance (AUPR value) and network size (the number of species) according to the network types: random networks (random), scale-free networks (sf), and small-world networks (sw). Competitive interaction matrices were considered. The cases of sparse networks ($\langle k \rangle = 2$; top panels) and dense networks ($\langle k \rangle = 8$; bottom panels) are shown. As representative examples, Pearson's correlation-based method (A and B), Pearson's partial correlation-based method (C and D), CCLasso (E and F), and SPEIC-EASI (G and H) are shown. s_{max} was set to 0.5. The number of samples was set to 300. Error bars indicate standard deviations.

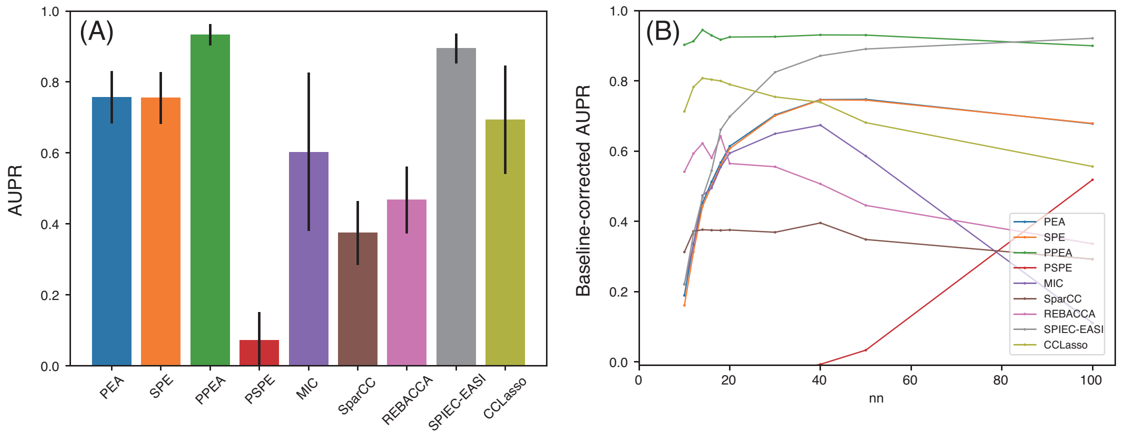

Figure S12: Differences in co-occurrence network performance between methods. (A) AUPR values for 50-node networks with average degree $\langle k \rangle = 2$. Error bars indicate standard deviations. (B) Relationship between the baseline-corrected AUPR value and network size n for $\langle k \rangle = 2$. Mutualistic interaction matrices and random network structure were considered. $s_{\max}$ was set to 0.5. The number of samples was set to 300.

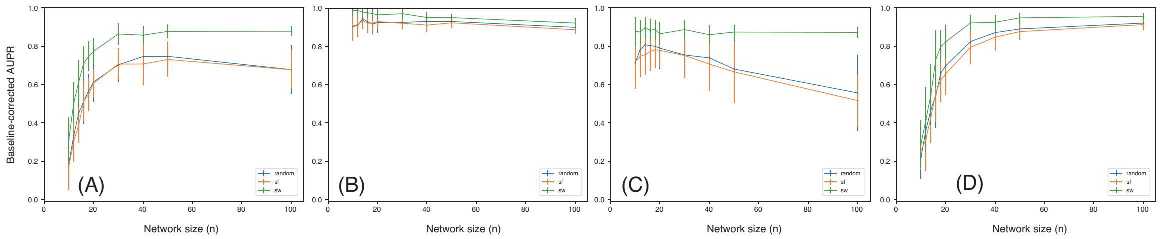

Figure S13: Relationships between co-occurrence network performance (AUPR value) and network size (the number of species) according to network type: random networks (random), scale-free networks (sf), and small-world networks (sw). Mutualistic interaction matrices were considered. Average degree $\langle k \rangle = 2$. As representative examples, Pearson's correlation-based method (A), Pearson's partial correlation-based method (B), CCLasso (C), and SPEIC-EASI (D) are shown. $s_{\max}$ was set to 0.5. The number of samples was set to 300. Error bars indicate standard deviations.

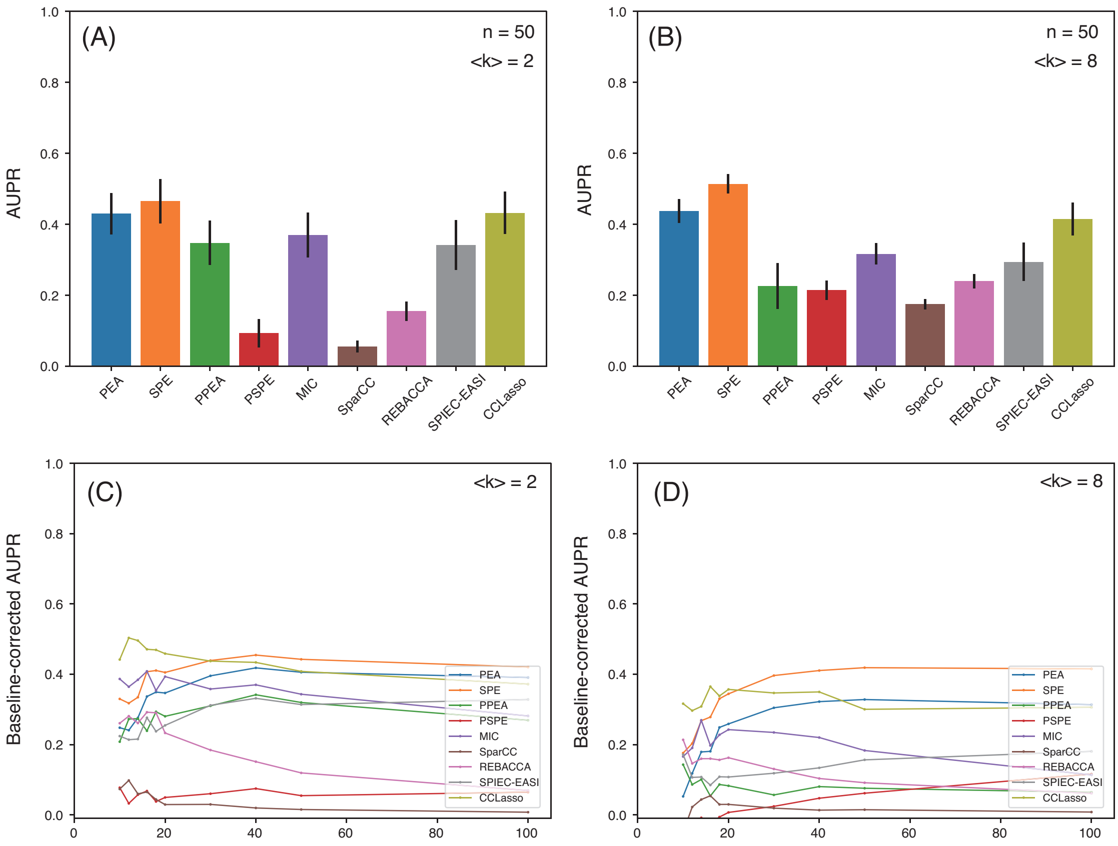

Figure S14: Differences in co-occurrence network performance between methods. AUPR values for 50-node networks with average degree $\langle k \rangle = 2$ (A) and $\langle k \rangle = 8$ (B). Error bars indicate standard deviations. Relationships between the baseline-corrected AUPR value and network size n for $\langle k \rangle = 2$ (C) and $\langle k \rangle = 8$ (D). Predator–prey (parasitic) interaction matrices and random network structure were considered. $s_{\max}$ was set to 0.5. The number of samples was set to 300.

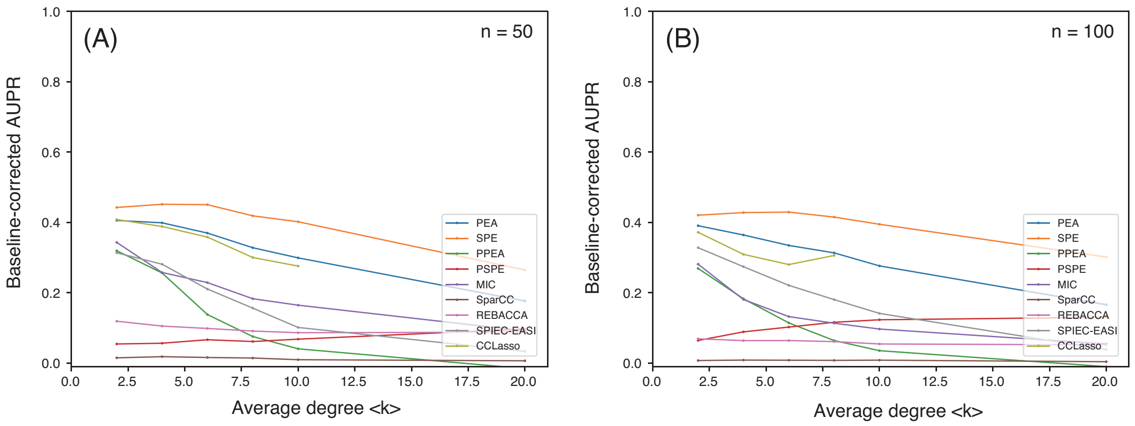

Figure S15: Relationships between co-occurrence network performance (baseline-corrected AUPR value) and average degree when network size $n = 50$ (A) and $n = 100$ (B). Predator–prey (parasitic) interaction matrices and random network structure were considered. s_{max} was set to 0.5. The number of samples was set to 300. The baseline-corrected AUPR values of CCLasso were not calculated when $\langle k \rangle > 10$ in (B) because of high computational costs.

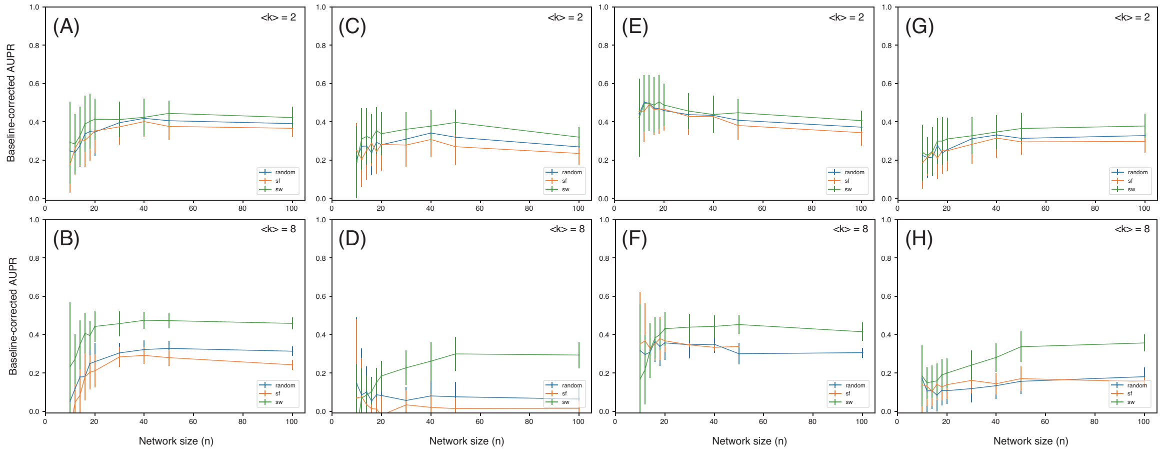

Figure S16: Relationships between co-occurrence network performance (AUPR value) and network size (the number of species) according to the network types: random networks (random), scale-free networks (sf), and small-world networks (sw). Predator–prey (parasitic) interaction matrices were considered. The cases of sparse networks ($\langle k \rangle = 2$; top panels) and dense networks ($\langle k \rangle = 8$; bottom panels) are shown. As representative examples, Pearson's correlation-based method (A and B), Pearson's partial correlation-based method (C and D), CCLasso (E and F), and SPEIC-EASI (G and H) are shown. s_{max} was set to 0.5. The number of samples was set to 300. The baseline-corrected AUPR values for scale-free networks of were not calculated when $n > 50$ in (F) due to high computational costs.

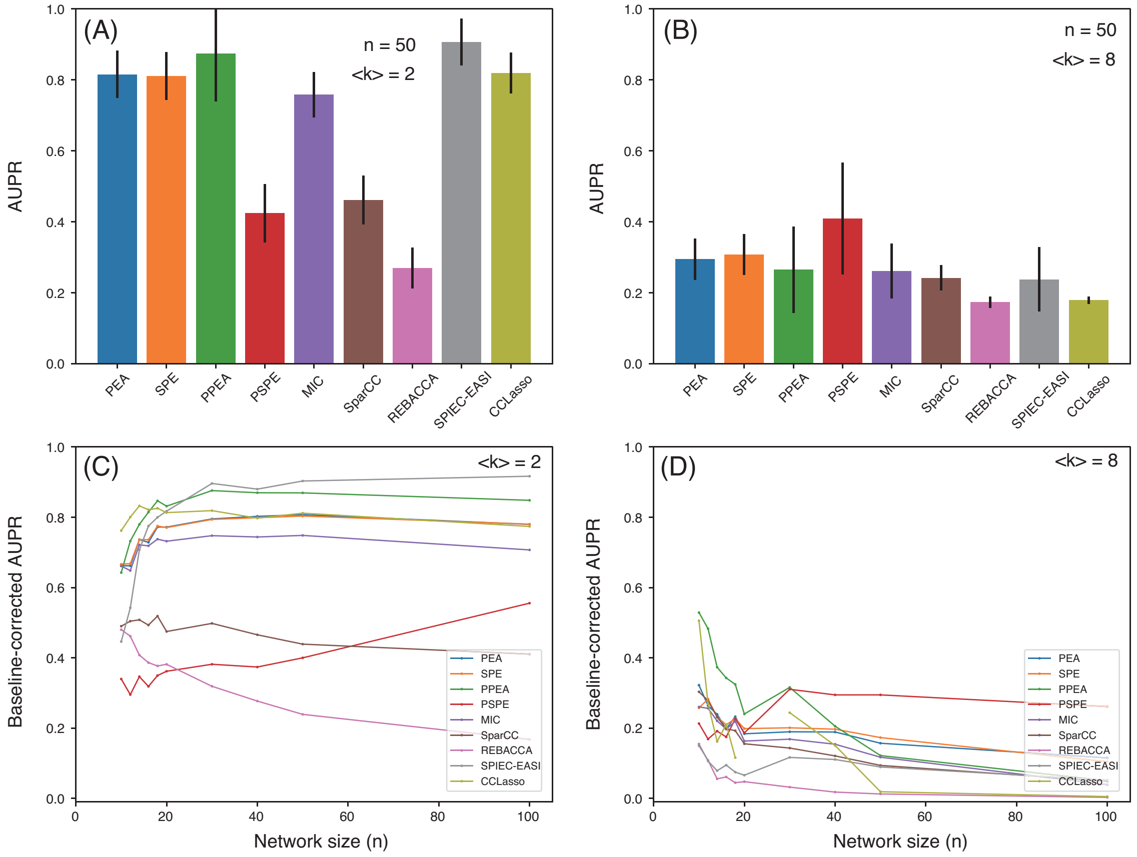

Figure S17: Differences in co-occurrence network performance between methods. AUPR values for 50-node networks with average degree $\langle k \rangle = 2$ (A) and $\langle k \rangle = 8$ (B). Error bars indicate standard deviations. Relationships between the baseline-corrected AUPR value and network size n for $\langle k \rangle = 2$ (C) and $\langle k \rangle = 8$ (D). Mutualism-competition mixture interaction matrices and random network structure were considered. $s_{\max}$ was set to 0.5. The number of samples was set to 300.

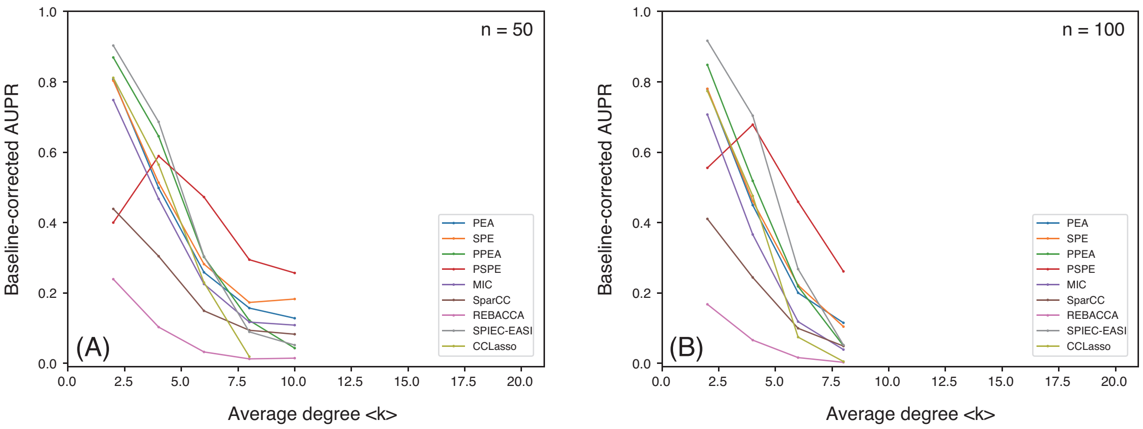

Figure S18: Relationships between co-occurrence network performance (baseline-corrected AUPR value) and average degree when network size n = 50 (A) and n = 100 (B). Mutualism-competition mixture interaction matrices and random network structure were considered. $s_{\max}$ was set to 0.5. The number of samples was set to 300.

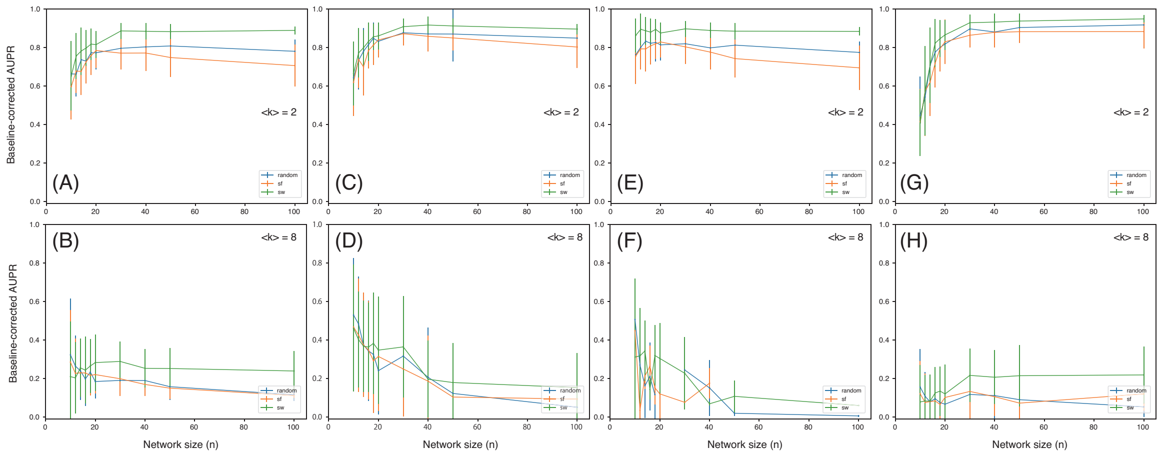

Figure S19: Relationships between co-occurrence network performance (AUPR value) and network size n according to the network types: random networks (random), scale-free networks (sf), and small-world networks (sw). Mutualism-competition mixture interaction matrices were considered. The cases of sparse networks ($\langle k \rangle = 2$; top panels) and dense networks ($\langle k \rangle = 8$; bottom panels) are shown. As representative examples, Pearson's correlation-based method (A and B), Pearson's partial correlation-based method (C and D), CCLasso (E and F), and SPEIC-EASI (G and H) are shown. $s_{\max}$ was set to 0.5. The number of samples was set to 300. The baseline-corrected AUPR values for scale-free networks of were not calculated when n > 40 in (F) due to high computational costs.

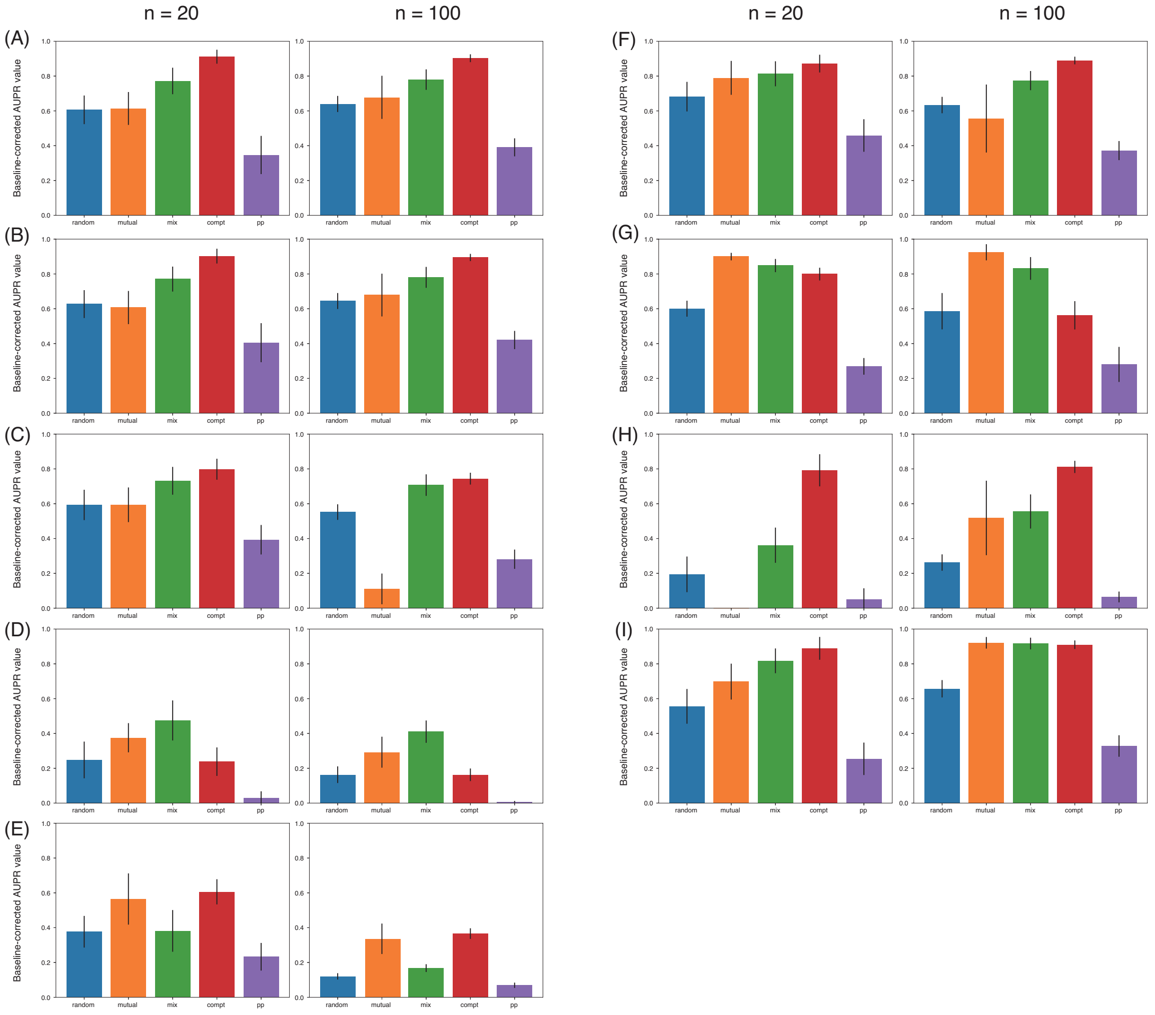

Figure S20: Effects of community type on co-occurrence network performance (baseline-corrected AUPR value) in Pearson's correlation-based method (A), Spearman's correlation-based method (B), MIC-based method (C), SparCC (D), REBACCA(E), CCLasso (F), Pearson's partial correlation-based method (G), Spearman's correlation-based method (H), and SPEIC-EASI (I). Vertical-axis labels correspond to the community types: random community (random), mutualistic community (mutual), competition–mutualism mixture community (mix), competitive community (compt), and predator–prey (parasitic) community (pp). The cases in which network size $n = 100$ and $n = 20$ are shown. Average degree $\langle k \rangle = 2$. Random network structure was considered. $s_{\max}$ was set to 0.5. The number of samples was set to 300. Error bars indicate standard deviations.

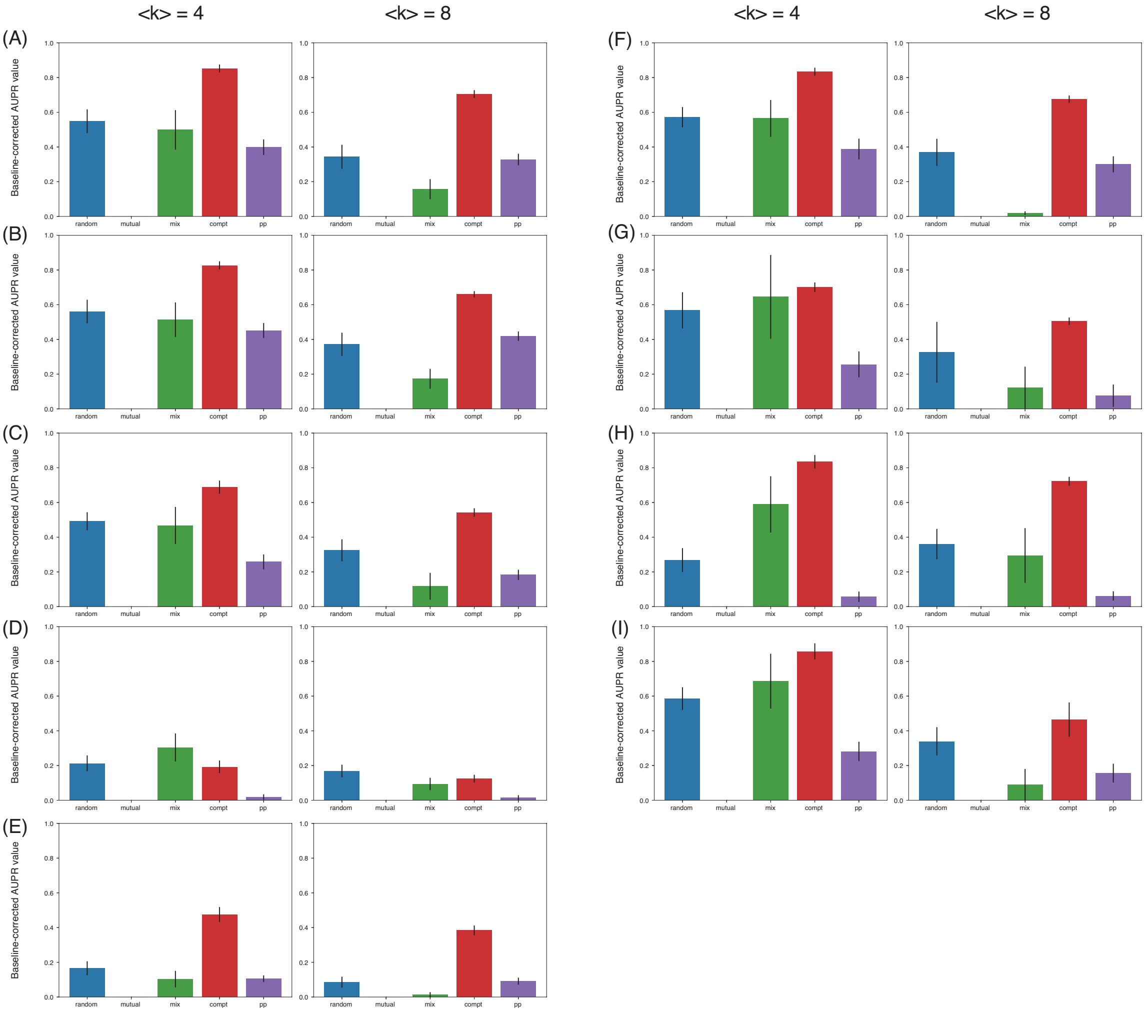

Figure S21: Effects of community type on co-occurrence network performance (baseline-corrected AUPR value) in Pearson's correlation-based method (A), Spearman's correlation-based method (B), MIC-based method (C), SparCC (D), REBACCA(E), CCLasso (F), Pearson's partial correlation-based method (G), Spearman's correlation-based method (H), and SPEIC-EASI (I). Vertical-axis labels correspond to the community types: random community (random), mutualistic community (mutual), competition–mutualism mixture community (mix), competitive community (compt), and predator–prey (parasitic) community (pp). The cases in which average degree $\langle k \rangle = 4$ and $\langle k \rangle = 8$ and network size $n = 100$ are shown. Random network structure was considered. $s_{\max}$ was set to 0.5. The number of samples was set to 300. The baseline-corrected AUPR values for mutualistic communities were not calculated because of numerical divergence in the simulation of the GLV equations. Error bars indicate standard deviations.

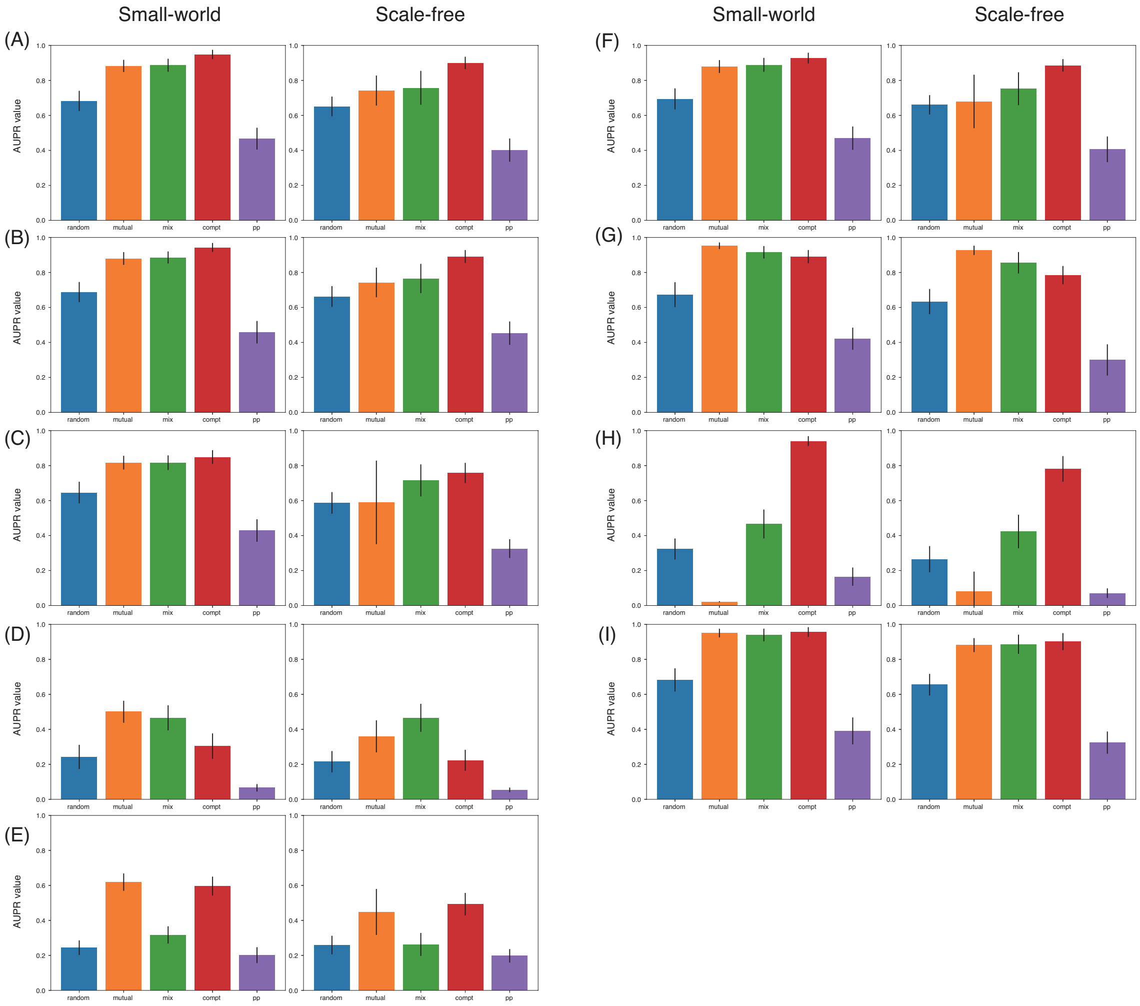

Figure S22: Effects of community type on co-occurrence network performance (baseline-corrected AUPR value) in Pearson's correlation-based method (A), Spearman's correlation-based method (B), MIC-based method (C), SparCC (D), REBACCA(E), CCLasso (F), Pearson's partial correlation-based method (G), Spearman's correlation-based method (H), and SPEIC-EASI (I). Vertical-axis labels correspond to the community types: random community (random), mutualistic community (mutual), competition–mutualism mixture community (mix), competitive community (compt), and predator–prey (parasitic) community (pp). The cases of small-world networks and scale-free networks are shown. Network size $n = 100$ and average degree $\langle k \rangle = 2$. Random network structure was considered. $s_{\max}$ was set to 0.5. The number of samples was set to 300. Error bars indicate standard deviations.
